# Burden of lip and oral cavity cancer among young people across South, East, and Southeast Asia: trends from 1990 to 2021 and predictions to 2030

**DOI:** 10.3389/fonc.2025.1680008

**Published:** 2026-01-16

**Authors:** Hui Chen, Meiling Hu, Meiling Feng, Xueru Chen, Xuan Guo, Jincai Guo

**Affiliations:** 1 Department of Pharmacy, Changsha Stomatological Hospital, Changsha, China; 2 School of Stomatology, Hunan University of Chinese Medicine, Changsha, China; 3 Department of Pharmacy, Hunan Aerospace Hospital, Changsha, Hunan, China; 4 School of Pharmacy, Hunan University of Chinese Medicine, Changsha, China

**Keywords:** deaths, disability-adjusted life years, disease burden, epidemiology, incidence, lip and oral cavity cancer

## Abstract

**Background:**

Lip and oral cavity cancer (LOC) is a major public health challenge in Asia. Nevertheless, a critical gap remains in understanding the epidemiological burden of LOC among young people (15–44 years) in the region. This study aims to analyze the burden and risk factors of LOC in this age group across the four Global Burden of Disease (GBD) Asian regions from 1990 to 2021 and projects trends to 2030.

**Methods:**

Data on the incidence, deaths, disability-adjusted life years (DALYs), and risk factors of LOC from 1990 to 2021 were obtained from the GBD 2021 study for East, South, Southeast Asia, and High-income Asia Pacific. This study assessed the LOC burden among young people (15–44 years) through age- and sex-stratified analyses, evaluated temporal trends via joinpoint regression, examined risk factor contributions, and projected trends to 2030 using the Nordpred age-period-cohort model.

**Results:**

From 1990 to 2021, the age-standardized incidence rate (ASIR) increased across all subregions, with the largest rise in East Asia. In contrast, age-standardized mortality rate (ASMR) and age-standardized DALYs rate declined everywhere except South Asia. In 2021, South Asia bore the heaviest LOC burden among young people in the four Asian subregions. India reported the highest incident cases, deaths, and DALYs in 2021, and Pakistan had the highest ASR for all three metrics. Taiwan (Province of China) showed the largest increase in ASRs over the period. In 2021, smoking had the highest contribution in East Asia, alcohol use in High-income Asia Pacific, and chewing tobacco in South Asia. Projections to 2030 indicate rising ASIR in East, South, and Southeast Asia but declines in High-income Asia Pacific; decreasing ASMR everywhere except South Asia; and increasing age-standardized DALYs rate in East and South Asia but decreases elsewhere.

**Conclusions:**

LOC imposes a substantial and growing burden on young people in South, East, and Southeast Asia, marked by rising ASIR since 1990 and projected increases through 2030. South Asia faces the most urgent challenge with concurrent rises in incidence, mortality, and DALYs—most notably in the 20–24 age group. Region-specific interventions targeting predominant risk factors are critically needed.

## 1 Introduction

Lip and oral cavity cancer (LOC) presents a significant and evolving global public health challenge. While traditionally associated with older populations, compelling evidence now confirms an alarming rise in incidence among younger individuals globally (1). A recent study showed that from 1990 to 2021, incidence, mortality, and disability-adjusted life-years (DALYs) rates of LOC and pharyngeal cancers grew among people aged 15–39 worldwide (2). This trend highlights the unique vulnerabilities of adolescence and early adulthood, as well as widening geographic and gender disparities. It signals a distinct clinical entity potentially reflecting complex gene-environment-behavior interactions in modern societies. Notably, the burden is most acute in Asia, where our prior analysis of Global Burden of Disease (GBD) 2021 data indicates that South and East Asian countries carry a disproportionately high LOC burden among younger populations (1). While existing studies, including the aforementioned global analysis of 15–39-year-olds and national or regional reports from China (3), India (4), Iran (5), Brazil (6), and other BRICS nations (7), have described the overall burden, most focus on broader age groups or national-level data, obscuring key subregional differences. For instance, a recent analysis by Lin et al. examined the spatiotemporal patterns and risk factors of LOC across all age groups in Asia (8). While their study provides important insights into the continent’s overall epidemic, it does not isolate the burden on young people—an age group with distinct risk factors and life-course considerations. As a result, the specific burden of early-onset LOC across Asian subregions is poorly understood, hindering the development of targeted prevention strategies.

To fill this gap, we analyzed GBD 2021 data, focusing on individuals aged 15–44 in East Asia, Southeast Asia, South Asia, and the High-income Asia Pacific. We report age-standardized incidence rate (ASIR), age-standardized mortality rate (ASMR), and age-standardized DALYs rate, enabling comparisons across regions and uncovering heterogeneity hidden in aggregate data. In addition to current estimates, we applied Nordpred age-period-cohort (APC) model to project trends through 2030 for this vulnerable group. This combined approach—capturing both current burden and future trajectories—offers novel insights into early-onset LOC in Asia. Our findings provide essential evidence to guide regionally tailored prevention strategies, inform health policy, and optimize resource allocation to reduce the growing burden among young people in high-risk Asian regions.

## 2 Materials and methods

### 2.1 Data sources

The GBD 2021 comprehensively evaluates health impairments of 371 diseases and injuries and 88 risk factors. Its methodology for disease burden estimation is detailed in previous studies (9, 10). Data on LOC incidence, deaths, DALYs, their corresponding age-standardized rate (ASR), and risk factors were extracted from the GBD 2021 study (https://vizhub.healthdata.org/gbd-results/). This study focused on young people aged 15–44 years (stratified by 5-year age groups from 15–19 to 40-44) across four Asian subregions: East Asia, South Asia, Southeast Asia, and High-income Asia Pacific. The age range of 15–44, defined as “young people” in recent GBD publications (11), was selected to capture the burden of early-onset LOC and represent the transition from adolescence to adulthood. A complete list of countries in each subregion is provided in **Supplementary Table 1**.

The Global Cancer Observatory (GLOBOCAN) 2022 project provides estimates for 36 cancer types across 185 countries and regions, including data on cancer incidence, mortality, and mortality-to-incidence ratios. To assess the reliability of the data used in this study, validation and consistency analysis was conducted against the corresponding estimates from GLOBOCAN 2022 (12). All GLOBOCAN 2022 data were sourced from the Cancer Today platform (https://gco.iarc.who.int/today/en/dataviz/tables). The research followed the Guidelines for Accurate and Transparent Health Estimates Reporting (13). As the data employed were publicly available, ethical review and informed consent were waived.

### 2.2 Definition of LOC

In the GBD 2021, LOC was defined using codes C00 to C08 in alignment with the corresponding International Classification of Diseases and Related Health Problems, Tenth Revision (ICD 10) (9).

### 2.3 Statistical analyses

#### 2.3.1 Descriptive analysis

The epidemiological burden of LOC among young people (15–44 years) in four Asian subregions was assessed from 1990 to 2021 through analysis of case counts and ASR of incidence, deaths, and DALYs, stratified by sex and age. All rates were expressed per 100,000 population, and the GBD 2021 world standard population was used for age standardization (9).

#### 2.3.2 Trend analysis

Investigating temporal trends in disease burden is crucial to epidemiology, facilitating the development of tailored preventive strategies. Joinpoint regression analysis is commonly employed in epidemiological research to assess such trends in incidence or mortality rates (14). In this study, this method was applied to analyze the temporal trends in the ASIR, ASMR, and age-standardized DALYs rate of LOC from 1990 to 2021. This analysis involved key steps: first, the Grid Search Method (GSM), the default approach in Joinpoint regression, is employed to identify all potential joinpoints for segmented interval functions. In this process, GSM divides the parameter space into grids, calculates the Sum of Squared Errors (SSE) and Mean Squared Error (MSE) for each scenario, and selects the grid point with the minimum MSE as the optimal joinpoint. Subsequently, the optimal number of joinpoints is determined using the Monte Carlo permutation test (see **Supplementary Methods**) (14). Detailed information on joinpoint regression methodology is available on its official website (https://surveillance.cancer.gov/joinpoint/). The model calculated the average annual percent change (AAPC). An AAPC >0 indicates an increasing trend in ASR, while AAPC <0 reflects a decreasing trend. If the AAPC is not statistically different from zero, the trend is considered stable. The model fit was evaluated through residual analysis, where an average relative error ≤10.00% between observed and fitted values indicated a good fit (15). Additionally, a sensitivity analysis was performed using the lower and upper bounds of the data to test the robustness of the findings against potential fluctuations.

#### 2.3.3 Attributable risk factors

This study further analyzed the proportion of LOC deaths and DALYs attributable to smoking, alcohol use, and chewing tobacco. Within the GBD 2021 framework, the selection of these risk factors was based on their risk-outcome pairs meeting the World Cancer Research Fund grades of convincing or probable evidence. The theoretical minimum risk exposure level was defined as lifelong non-use for smoking and chewing tobacco, while for alcohol use, it was defined as a daily intake distribution ranging from 0 to 10 grams (10). Additionally, the AAPC was employed to assess the temporal trends in the contribution of these risk factors from 1990 to 2021.

#### 2.3.4 Prediction

The Nordpred APC model was used to predict future trends in the burden of LOC among young people across four Asian subregions, including projections of incidence, death, and DALY counts and ASR from 2022 to 2030. Population data for projections were obtained from the 2024 revision of the World Population Prospects (https://population.un.org/wpp/). The reliability of the Nordpred model has been validated in previous studies (16, 17). Sensitivity analysis and residual analysis were performed as described in section 2.3.2 to evaluate model robustness and fit.

#### 2.3.5 Software and statistical tools

Statistical analyses were performed using R software (version 4.4.1) with the “Nordpred” package for age-period-cohort modeling and the “ggplot2” package for data visualization, as well as Joinpoint regression software (version 5.2.0) for trend analysis.

## 3 Results

### 3.1 Regional burden and trends of LOC in young people

**Figure 1:**
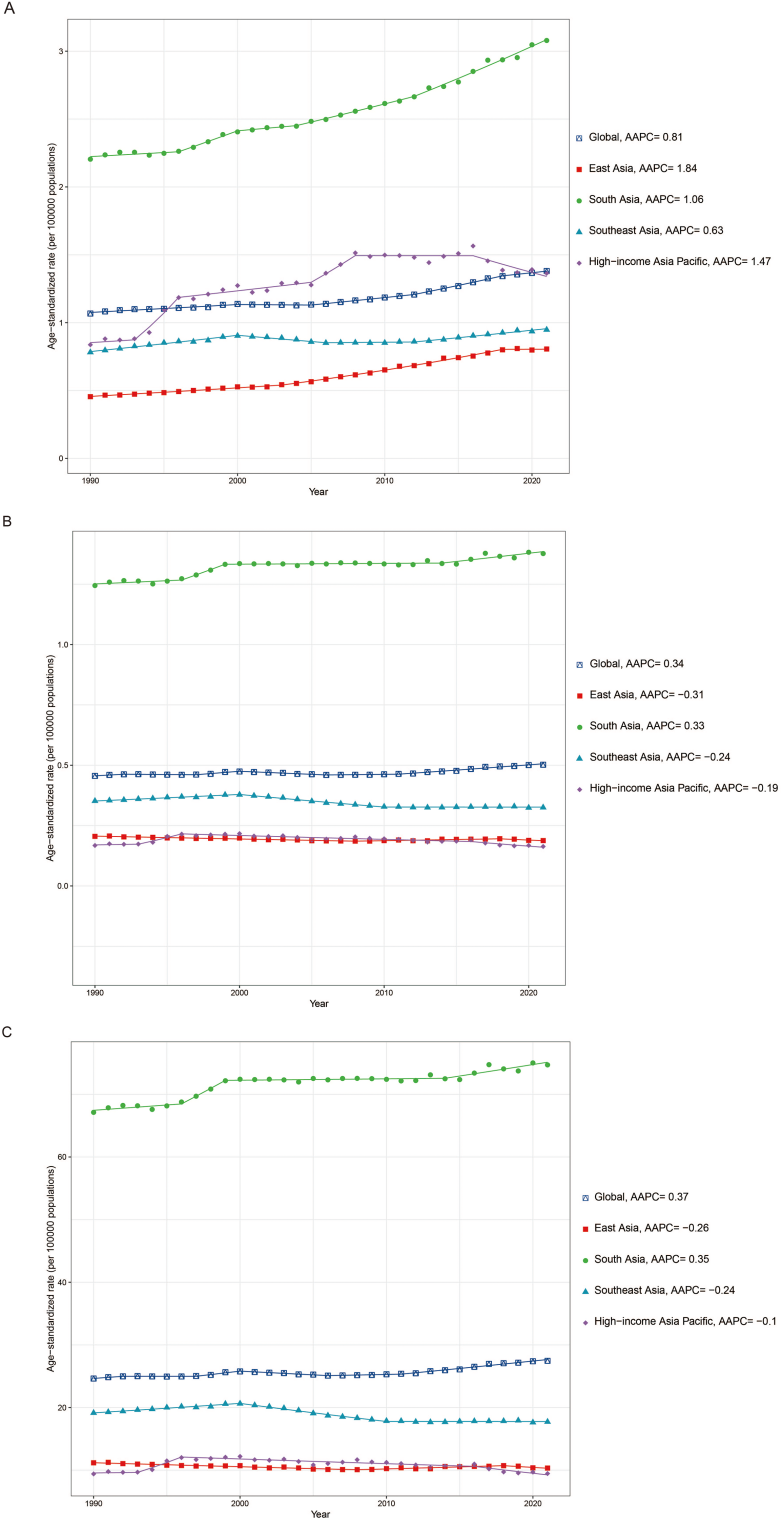
The temporal trends of the age-standardized rate of incidence **(A)**, deaths **(B)**, and DALYs **(C)** of lip and oral cavity cancer among young people across four Asian subregions. AAPC, average annual percent change; DALYs, age-standardized disability-adjusted life years.

In 2021, South Asia had the heaviest LOC burden among young populations across the four Asian subregions, with 26,729 incident cases, 11,922 deaths, and 650,469 DALYs, accounting for over half of the corresponding global totals. Its ASIR, ASMR, and age-standardized DALYs rate all exceeded twice the global averages. Conversely, High-income Asia Pacific recorded the lowest case numbers and ASR for deaths and DALYs, and East Asia had the lowest ASIR. From 1990 to 2021, the ASIR demonstrated increasing trends across all subregions, with East Asia exhibiting the most pronounced increase. In contrast, the ASMR and age-standardized DALYs rate showed declining trends in most subregions, except for South Asia (**Figure 1**, **Tables 1**–**3**).

**Table 1:** Number of cases and ASR of LOC incidence among young people in 1990 and 2021, at global, Asian four regional and national level, along with AAPC from 1990 to 2021.

Location	1990		2021		AAPC (95%CI) 1990–2021	*P*
	Number (95%UI)	ASR (95%UI)	Number (95%UI)	ASR (95%UI)		
Global	23825(22320-25349)	1.07(1-1.13)	49210(42632-54947)	1.38(1.2-1.54)	0.81 (0.75 to 0.85)	<0.001
Male	15415(14144-16619)	1.37(1.26-1.48)	30179(25273-34074)	1.67(1.4-1.89)	0.67 (0.63 to 0.71)	<0.001
Female	8410(7724-9200)	0.75(0.69-0.82)	19031(16286-22404)	1.08(0.93-1.28)	1.19 (1.14 to 1.22)	<0.001
Regions						
East Asia	2584(2220-2978)	0.45(0.39-0.52)	5409(4491-6440)	0.81(0.67-0.96)	1.84 (1.75 to 1.92)	<0.001
High-income Asia Pacific	716(642-803)	0.84(0.75-0.94)	1023(872-1198)	1.36(1.16-1.6)	1.47 (1.24 to 1.63)	<0.001
South Asia	9315(8132-10553)	2.2(1.93-2.49)	26729(21132-31786)	3.08(2.44-3.65)	1.06 (1.02 to 1.1)	<0.001
Southeast Asia	1449(1205-1714)	0.78(0.65-0.92)	3217(2673-3859)	0.95(0.79-1.14)	0.63 (0.58 to 0.68)	<0.001
Countries						
Bangladesh	670(406-1025)	1.8(1.1-2.75)	1539(730-2597)	2.02(0.96-3.4)	0.43 (0.37 to 0.48)	<0.001
Bhutan	4(2-6)	1.69(0.94-2.66)	8(4-13)	1.97(1.03-3.4)	0.45 (0.39 to 0.54)	<0.001
Brunei Darussalam	2(1-3)	1.47(1-2.11)	4(3-5)	1.47(1.02-2.04)	0.08 (-0.02 to 0.18)	0.092
Cambodia	18(11-29)	0.51(0.31-0.81)	57(36-89)	0.72(0.46-1.13)	1.12 (1.1 to 1.14)	<0.001
China	2282(1924-2666)	0.41(0.35-0.48)	4535(3648-5574)	0.7(0.57-0.86)	1.74 (1.6 to 1.81)	<0.001
Democratic People's Republic of Korea	46(27-73)	0.52(0.31-0.83)	74(44-123)	0.58(0.34-0.97)	0.36 (0.33 to 0.38)	<0.001
India	6766(5967-7705)	1.99(1.75-2.26)	17948(14322-21224)	2.65(2.12-3.13)	0.89 (0.83 to 0.94)	<0.001
Indonesia	388(285-504)	0.52(0.38-0.67)	832(610-1124)	0.59(0.43-0.8)	0.45 (0.43 to 0.47)	<0.001
Japan	575(509-652)	0.94(0.83-1.06)	754(639-895)	1.59(1.35-1.89)	1.55 (1.08 to 1.95)	<0.001
Lao People's Democratic Republic	7(4-12)	0.51(0.3-0.88)	20(12-30)	0.58(0.35-0.89)	0.39 (0.37 to 0.42)	<0.001
Malaysia	68(45-101)	0.92(0.61-1.36)	187(123-277)	1.17(0.77-1.73)	0.75 (0.64 to 0.86)	<0.001
Maldives	0(0-1)	0.56(0.24-1.06)	2(1-3)	0.58(0.38-0.84)	0.1 (-0.1 to 0.28)	0.323
Mauritius	5(4-5)	0.89(0.75-1.04)	7(5-8)	1.07(0.88-1.29)	0.85 (0.54 to 1.17)	<0.001
Myanmar	87(46-150)	0.55(0.29-0.95)	143(86-221)	0.56(0.34-0.86)	0.05 (0.02 to 0.08)	0.003
Nepal	104(67-160)	1.5(0.96-2.31)	260(158-406)	1.93(1.18-3.01)	0.82 (0.79 to 0.84)	<0.001
Pakistan	1772(1311-2357)	4.76(3.55-6.3)	6976(4854-9707)	6.89(4.82-9.54)	1.19 (1.15 to 1.22)	<0.001
Philippines	195(164-229)	0.8(0.67-0.94)	349(288-426)	0.68(0.56-0.83)	-0.6 (-0.64 to -0.55)	<0.001
Republic of Korea	127(95-169)	0.58(0.43-0.77)	241(164-346)	1.01(0.69-1.45)	1.84 (1.77 to 1.92)	<0.001
Seychelles	1(0-1)	1.94(1.27-2.86)	1(1-2)	2.7(1.69-4.12)	1.21 (0.96 to 1.44)	<0.001
Singapore	12(10-15)	0.69(0.56-0.86)	24(19-31)	0.76(0.59-0.97)	0.63 (0.07 to 1.05)	0.034
Sri Lanka	99(65-145)	1.26(0.82-1.86)	184(99-300)	1.8(0.97-2.91)	1.13 (0.82 to 1.35)	<0.001
Taiwan (Province of China)	256(211-307)	2.63(2.17-3.15)	800(622-1015)	6.28(4.88-7.96)	2.87 (2.71 to 3.03)	<0.001
Thailand	342(232-490)	1.34(0.91-1.91)	714(473-1007)	2.29(1.52-3.24)	1.81 (1.71 to 1.91)	<0.001
Timor-Leste	1(1-2)	0.34(0.2-0.54)	2(1-4)	0.45(0.27-0.71)	0.78 (0.68 to 0.88)	<0.001
Viet Nam	235(155-346)	1(0.67-1.48)	714(465-1097)	1.41(0.92-2.16)	1.12 (1.09 to 1.14)	<0.001

AAPC, average annual percent change; ASR, age-standardized rate; GBD, Global Burden of Disease.

**Table 2:** Number of cases and ASR of LOC mortality among young people in 1990 and 2021, at global, Asian four regional and national level, along with AAPC from 1990 to 2021.

Location	1990		2021		AAPC (95%CI) 1990–2021	*P*
	Number (95%UI)	ASR (95%UI)	Number (95%UI)	ASR (95%UI)		
Global	10161(9365-10969)	0.46(0.42-0.49)	17879(15060-20328)	0.5(0.42-0.57)	0.34 (0.31 to 0.36)	<0.001
Male	6826(6118-7535)	0.61(0.55-0.67)	11724(9523-13480)	0.65(0.53-0.75)	0.21 (0.18 to 0.25)	<0.001
Female	3335(3014-3720)	0.3(0.27-0.33)	6155(5131-7489)	0.35(0.29-0.43)	0.55 (0.46 to 0.6)	<0.001
Regions						<0.001
East Asia	1159(991-1338)	0.21(0.18-0.24)	1261(1050-1520)	0.19(0.16-0.23)	-0.31 (-0.41 to -0.22)	<0.001
High-income Asia Pacific	145(136-156)	0.17(0.16-0.18)	126(116-137)	0.16(0.15-0.18)	-0.19 (-0.52 to 0.12)	0.163
South Asia	5238(4570-5943)	1.24(1.09-1.41)	11922(9430-14205)	1.38(1.09-1.64)	0.33 (0.29 to 0.37)	<0.001
Southeast Asia	647(538-763)	0.35(0.29-0.41)	1106(918-1319)	0.33(0.27-0.39)	-0.24 (-0.27 to -0.2)	<0.001
Countries						
Bangladesh	393(240-600)	1.06(0.65-1.62)	625(299-1075)	0.82(0.39-1.41)	-0.77 (-0.83 to -0.73)	<0.001
Bhutan	2(1-3)	1(0.55-1.58)	3(2-6)	0.85(0.43-1.46)	-0.56 (-0.61 to -0.51)	<0.001
Brunei Darussalam	1(0-1)	0.54(0.38-0.77)	1(1-1)	0.4(0.29-0.55)	-0.87 (-0.96 to -0.77)	<0.001
Cambodia	10(6-16)	0.29(0.17-0.46)	25(16-40)	0.32(0.2-0.51)	0.34 (0.32 to 0.37)	<0.001
China	1063(895-1244)	0.19(0.16-0.23)	1062(852-1310)	0.16(0.13-0.2)	-0.58 (-0.69 to -0.51)	<0.001
Democratic People's Republic of Korea	19(11-31)	0.22(0.13-0.35)	26(15-43)	0.2(0.12-0.33)	-0.26 (-0.27 to -0.25)	<0.001
India	3789(3334-4321)	1.12(0.98-1.27)	7801(6175-9252)	1.16(0.92-1.37)	0.05 (0 to 0.1)	0.064
Indonesia	192(143-246)	0.26(0.19-0.33)	343(248-464)	0.24(0.18-0.33)	-0.19 (-0.2 to -0.17)	<0.001
Japan	102(100-105)	0.16(0.16-0.17)	91(88-94)	0.19(0.18-0.19)	0.28 (-0.06 to 0.56)	0.089
Lao People's Democratic Republic	4(3-7)	0.31(0.18-0.52)	9(6-14)	0.28(0.17-0.42)	-0.37 (-0.39 to -0.34)	<0.001
Malaysia	28(19-41)	0.39(0.25-0.56)	57(38-83)	0.36(0.24-0.52)	-0.29 (-0.43 to -0.16)	<0.001
Maldives	0(0-0)	0.25(0.11-0.48)	1(0-1)	0.16(0.1-0.24)	-1.31 (-1.59 to -1.05)	<0.001
Mauritius	2(2-2)	0.34(0.3-0.38)	2(2-2)	0.35(0.3-0.4)	0.33 (-0.02 to 0.75)	0.063
Myanmar	48(25-82)	0.3(0.16-0.52)	61(37-94)	0.24(0.14-0.37)	-0.76 (-0.78 to -0.74)	<0.001
Nepal	62(40-96)	0.89(0.58-1.39)	116(71-181)	0.87(0.53-1.35)	-0.07 (-0.09 to -0.05)	<0.001
Pakistan	992(744-1295)	2.68(2.02-3.48)	3376(2383-4610)	3.35(2.38-4.56)	0.75 (0.72 to 0.78)	<0.001
Philippines	88(76-101)	0.36(0.32-0.42)	145(121-171)	0.28(0.24-0.33)	-0.84 (-0.9 to -0.79)	<0.001
Republic of Korea	39(30-50)	0.18(0.14-0.23)	30(22-42)	0.12(0.09-0.17)	-1.15 (-1.23 to -1.07)	<0.001
Seychelles	0(0-0)	0.88(0.58-1.31)	1(0-1)	1.01(0.63-1.5)	0.54 (0.26 to 0.79)	0.004
Singapore	3(2-3)	0.17(0.14-0.2)	3(3-4)	0.1(0.08-0.12)	-1.42 (-1.82 to -0.79)	0.002
Sri Lanka	41(27-59)	0.52(0.34-0.75)	53(29-86)	0.51(0.28-0.83)	0.01 (-0.29 to 0.23)	0.980
Taiwan (Province of China)	76(65-88)	0.79(0.68-0.92)	173(142-208)	1.33(1.09-1.6)	1.67 (1.5 to 1.83)	<0.001
Thailand	132(88-187)	0.52(0.35-0.74)	192(128-270)	0.6(0.4-0.85)	0.54 (0.43 to 0.65)	<0.001
Timor-Leste	1(0-1)	0.19(0.11-0.3)	1(1-2)	0.21(0.13-0.34)	0.23 (0.12 to 0.33)	<0.001
Viet Nam	101(68-147)	0.44(0.3-0.64)	215(138-334)	0.42(0.27-0.66)	-0.14 (-0.16 to -0.13)	<0.001

AAPC, average annual percent change; ASR, age-standardized rate; GBD, Global Burden of Disease.

**Table 3:** Number of cases and ASR of LOC DALYs among young people in 1990 and 2021, at global, Asian four regional and national level, along with AAPC from 1990 to 2021.

Location	1990		2021		AAPC (95%CI) 1990–2021	*P*
	Number (95%UI)	ASR (95%UI)	Number (95%UI)	ASR (95%UI)		
Global	555359(511854-599945)	24.64(22.72-26.59)	975629(816791-1111769)	27.46(22.97-31.32)	0.37 (0.34 to 0.4)	<0.001
Male	367910(329617-406845)	32.44(29.1-35.81)	630700(511034-725878)	35.06(28.39-40.36)	0.25 (0.21 to 0.29)	<0.001
Female	187448(169036-209074)	16.55(14.94-18.43)	344928(285126-421936)	19.73(16.28-24.17)	0.59 (0.51 to 0.65)	<0.001
Regions						
East Asia	63950(54601-73800)	11.18(9.54-12.9)	68723(57155-82691)	10.34(8.59-12.44)	-0.26 (-0.37 to -0.18)	0.000
High-income Asia Pacific	8056(7507-8687)	9.41(8.75-10.17)	7111(6510-7803)	9.47(8.67-10.4)	-0.1 (-0.48 to 0.24)	0.385
South Asia	286919(250063-325639)	67.12(58.56-76.09)	650469(511181-780262)	74.7(58.85-89.43)	0.35 (0.31 to 0.39)	<0.001
Southeast Asia	35953(29894-42467)	19.15(15.95-22.54)	60033(49847-71557)	17.72(14.72-21.12)	-0.24 (-0.27 to -0.2)	<0.001
Countries						
Bangladesh	21732(13222-33141)	57.25(34.97-87.27)	34498(16394-59506)	45.18(21.52-77.76)	-0.7 (-0.76 to -0.66)	<0.001
Bhutan	118(65-187)	53.65(29.75-85.13)	180(92-311)	46.02(23.49-79.42)	-0.52 (-0.57 to -0.46)	<0.001
Brunei Darussalam	39(27-55)	30.11(20.96-42.46)	58(42-80)	22.21(16-30.46)	-0.86 (-0.97 to -0.74)	<0.001
Cambodia	565(337-909)	15.73(9.45-25.21)	1378(873-2174)	17.47(11.09-27.53)	0.35 (0.32 to 0.37)	<0.001
China	58728(49423-68635)	10.6(8.91-12.39)	58272(46791-71619)	9.13(7.33-11.22)	-0.52 (-0.64 to -0.44)	<0.001
Democratic People's Republic of Korea	1070(630-1713)	12.05(7.11-19.26)	1421(822-2328)	11.17(6.46-18.29)	-0.25 (-0.26 to -0.24)	<0.001
India	206616(181389-235959)	60.11(52.85-68.57)	421252(332169-501583)	62.14(49.09-73.9)	0.05 (0 to 0.09)	0.060
Indonesia	10669(7933-13727)	14.07(10.51-18.05)	18659(13514-25267)	13.29(9.63-18)	-0.18 (-0.19 to -0.16)	<0.001
Japan	5655(5478-5842)	9.13(8.84-9.43)	5158(4957-5387)	10.84(10.41-11.32)	0.39 (0.07 to 0.66)	0.025
Lao People's Democratic Republic	241(139-405)	16.82(9.82-28.14)	519(313-796)	15.07(9.1-23.08)	-0.33 (-0.35 to -0.3)	<0.001
Malaysia	1560(1033-2249)	20.86(13.76-30.15)	3095(2067-4509)	19.37(12.92-28.23)	-0.29 (-0.44 to -0.16)	<0.001
Maldives	9(4-17)	13.73(6.02-26.18)	32(20-46)	8.85(5.72-12.93)	-1.28 (-1.53 to -1.02)	<0.001
Mauritius	94(83-106)	18.31(16.17-20.57)	117(101-134)	19(16.43-21.68)	0.12 (-0.19 to 0.59)	0.528
Myanmar	2662(1407-4555)	16.46(8.73-28.32)	3360(2041-5143)	13.08(7.96-20.02)	-0.75 (-0.77 to -0.72)	<0.001
Nepal	3393(2197-5237)	48.07(31.1-74.43)	6401(3886-9987)	47.14(28.72-73.33)	-0.05 (-0.08 to -0.03)	<0.001
Pakistan	55060(41149-72076)	145.04(108.95-189.07)	188138(132207-258110)	184.08(129.92-251.5)	0.79 (0.76 to 0.81)	<0.001
Philippines	4904(4254-5654)	19.9(17.26-22.9)	7970(6695-9383)	15.5(13.01-18.25)	-0.85 (-0.91 to -0.79)	<0.001
Republic of Korea	2201(1697-2814)	10(7.71-12.76)	1715(1217-2390)	7.14(5.05-9.97)	-1.02 (-1.12 to -0.93)	<0.001
Seychelles	13(8-19)	46.98(30.76-69.81)	29(18-43)	53(33.03-79.13)	0.57 (0.28 to 0.83)	0.004
Singapore	161(135-190)	9.47(7.93-11.19)	179(148-214)	5.74(4.74-6.87)	-1.19 (-1.6 to -0.58)	0.003
Sri Lanka	2257(1483-3269)	28.64(18.81-41.51)	2871(1581-4643)	27.94(15.51-45.09)	-0.06 (-0.39 to 0.18)	0.509
Taiwan (Province of China)	4152(3565-4834)	42.72(36.71-49.7)	9030(7470-10880)	70.47(58.27-84.94)	1.6 (1.43 to 1.77)	<0.001
Thailand	7245(4851-10312)	28.11(18.9-39.9)	10306(6917-14593)	33(22.16-46.84)	0.55 (0.45 to 0.67)	<0.001
Timor-Leste	33(19-51)	10.58(6.12-16.51)	59(36-93)	11.54(6.95-18.3)	0.23 (0.11 to 0.34)	0.000
Viet Nam	5647(3771-8173)	23.86(16.01-34.46)	11556(7434-17913)	22.98(14.8-35.54)	-0.11 (-0.12 to -0.09)	<0.001

AAPC, average annual percent change; ASR, age-standardized rate; DALYs, disability-adjusted life-years; GBD, Global Burden of Disease.

The residual analysis confirmed an excellent model fit, with average relative errors consistently below 10.00% (**Supplementary Table 2**). Furthermore, sensitivity analyses using the lower and upper bounds of the data produced similar trend patterns (**Supplementary Table 3**). These findings confirmed the reliability and robustness of the trend analysis methods.

### 3.2 National burden and trends of LOC in young people

At the national level across the four Asian subregions, India had the highest case numbers of LOC incidence, deaths, and DALYs among young people in 2021, followed by Pakistan. Seychelles reported the lowest counts for these indicators. Pakistan exhibited the highest ASIR, ASMR, and age-standardized DALYs rate, while Timor-Leste showed the lowest ASIR and Singapore demonstrated the lowest ASMR and age-standardized DALYs rate. From 1990 to 2021, Taiwan (Province of China) experienced the most pronounced increases in ASIR, ASMR, and age-standardized DALYs rate. Conversely, Philippines demonstrated the largest decrease in ASIR, Singapore in ASMR, and Maldives in age-standardized DALYs rate (**Tables 1**–**3**). According to GLOBOCAN 2022, India recorded the highest incidence and mortality cases of LOC among young people in 2022 (**Supplementary Table 4**), followed by Pakistan. The two countries also ranked first and second, respectively, in both ASIR and ASMR. These findings are consistent with the results from GBD 2021. Furthermore, the countries with a relatively low burden were similar across both databases (**Supplementary Table 4**).

### 3.3 Regional sex- and age-specific burden and trends

Globally, young men had higher numbers and ASR of LOC incidence, deaths, and DALYs than women. This gender disparity persisted across four Asian subregions, particularly in East Asia, where men’s ASR was more than three-fold higher than women’s (**Supplementary Table 5**). Temporal trends revealed distinct subregional patterns in gender-specific LOC burden. In East Asia, men showed upward trends in ASIR, ASMR, and age-standardized DALYs rate, while women had declining ASMR and age-standardized DALYs rate. Conversely, in the High-income Asia Pacific, women experienced rising trends in all three metrics, whereas men exhibited decreasing trends in ASMR and age-standardized DALYs rate. In South Asia, both genders exhibited increases in ASR for all three metrics. Uniform trends were observed in Southeast Asia, with rising ASIR but declining ASMR and age-standardized DALYs rate for both sexes.

Across the four Asian subregions, both the number of cases and ASR of LOC incidence, deaths, and DALYs among individuals aged 15–44 years increased with age, consistent with global patterns (**Supplementary Tables 6**–**8**). From 1990 to 2021, the ASIR showed a rising trend across all age subgroups in each of the four subregions. For ASMR and age-standardized DALYs rate, East Asia and Southeast Asia exhibited decreasing trends across all age groups, with the most significant decrease in the 15–19 age group. High-income Asia Pacific showed consistent ASMR and age-standardized DALYs rate trends across age groups, with some increasing and others decreasing; the largest rise was in the 35–39 age group and the steepest decrease was in the 15–19 age group. In contrast, South Asia experienced increasing trends in both ASMR and age-standardized DALYs rate across all age groups, with the most pronounced rise in the 20–24 age group.

### 3.4 Risk factors

**Figure 2:**
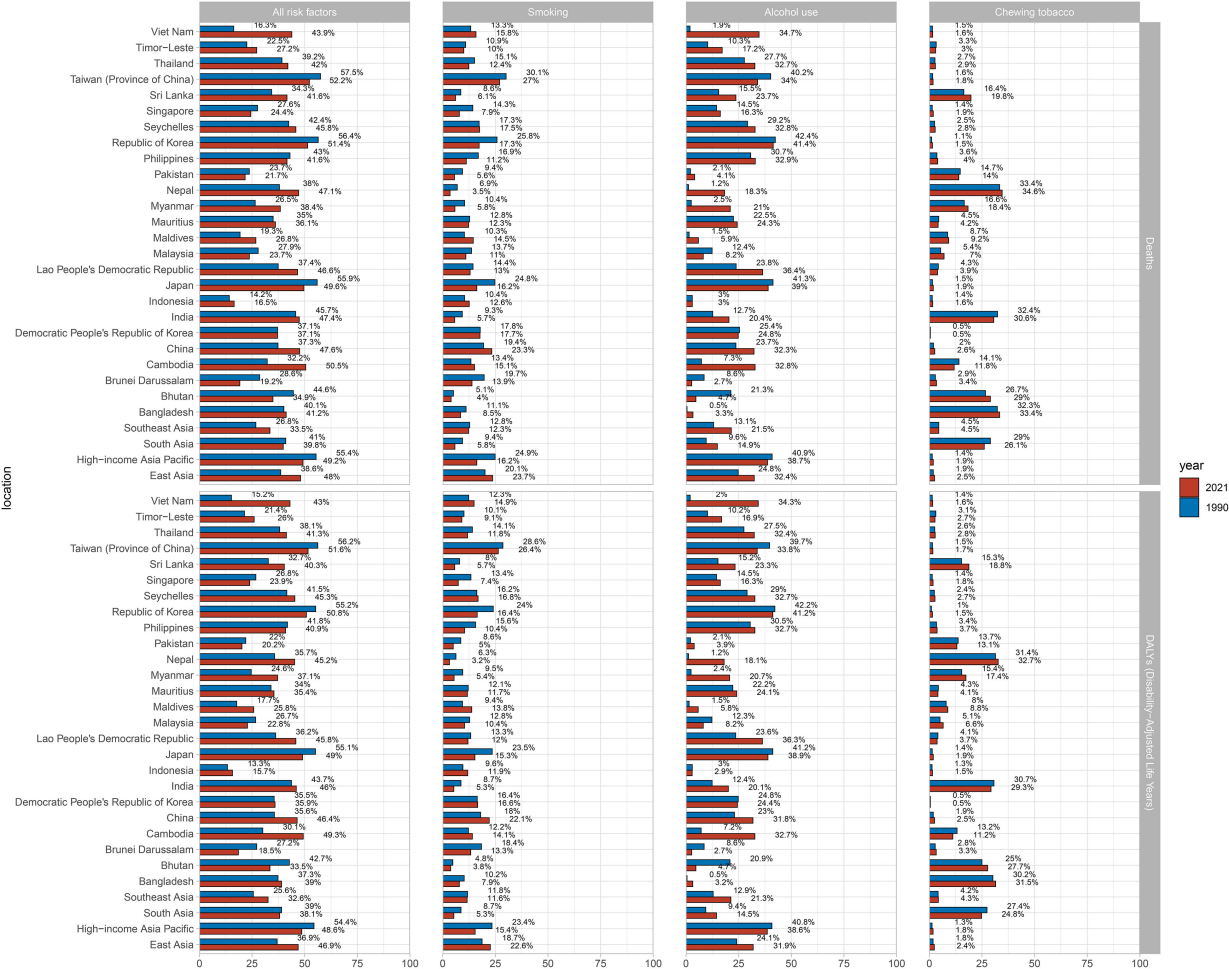
Comparison of attributable risk factors for lip and oral cavity cancer deaths and DALYs among young people, 1990 and 2021. DALYs: age-standardized disability-adjusted life years.

From 1990 to 2021, the overall contribution of all risk factors to LOC deaths and DALYs in young people increased in East Asia and Southeast Asia, while decreasing in High-income Asia Pacific and South Asia (**Figure 2**). Regarding specific risk factors, the contribution of smoking declined in all subregions except East Asia, while alcohol use increased in all subregions except High-income Asia Pacific. Chewing tobacco slightly increased in most regions but declined in South Asia. In 2021, the distribution of risk factor contributions to LOC deaths and DALYs among young populations varied significantly across the four subregions. Smoking had the highest contribution in East Asia, alcohol use in High-income Asia Pacific, and chewing tobacco in South Asia. Notably, alcohol use was the primary attributable risk factor for LOC deaths and DALYs among young populations in East Asia, High-income Asia Pacific, and Southeast Asia, whereas chewing tobacco predominant factor in South Asia. Across most studied countries, the contribution of smoking to LOC deaths and DALYs decreased, while the contributions of alcohol use and chewing tobacco increased. In 2021, smoking accounted for the largest proportion in China and Taiwan (Province of China). Alcohol use contributed over 30% in ten countries, with the highest in the Republic of Korea. Chewing tobacco’s contribution remained below 5% in most countries but reached approximately 30% in Bangladesh, India, and Nepal, peaking in Nepal.

**Figure 3:**
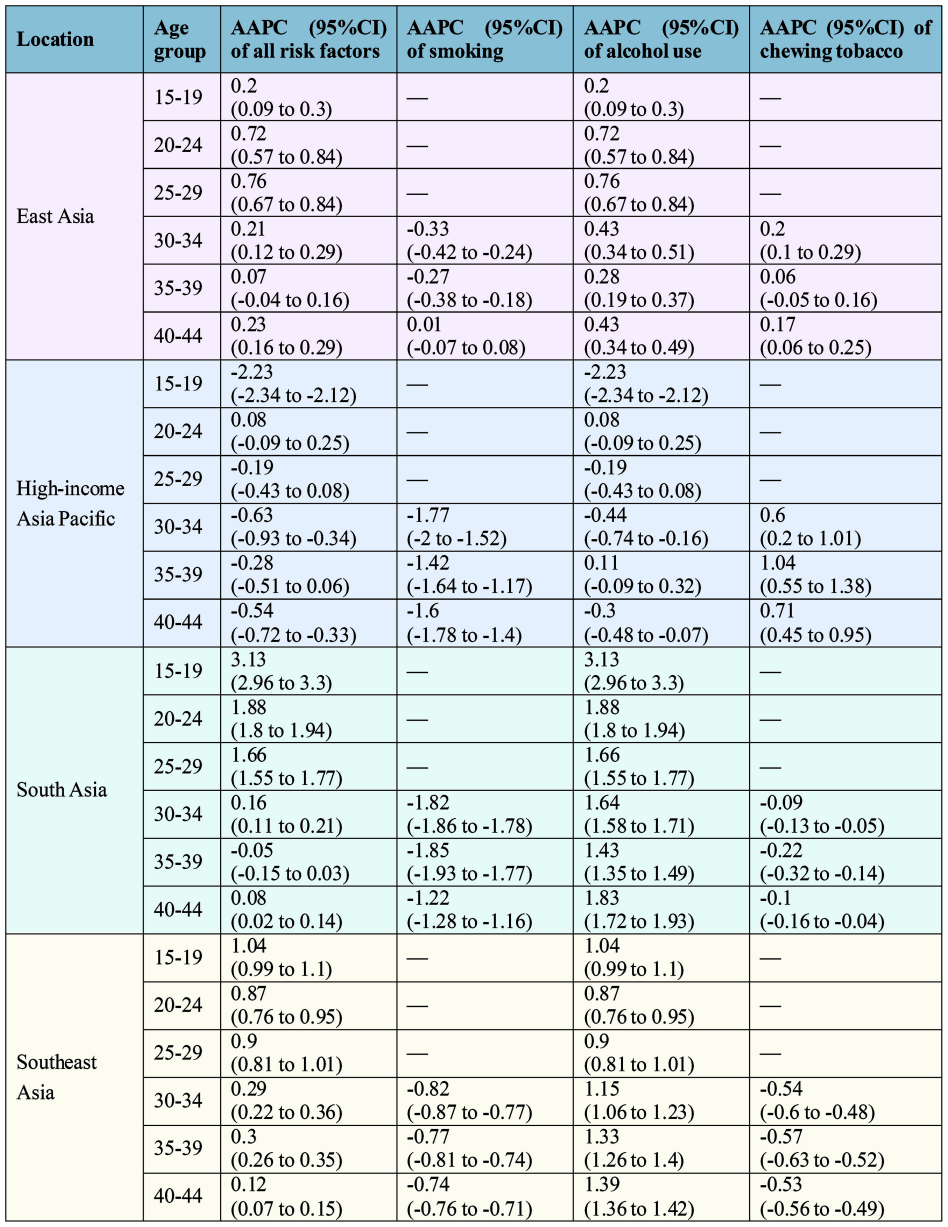
AAPC of lip and oral cavity cancer ASMR attributable to each risk factor among young people (1990–2021), by age group. AAPC, average annual percent change; ASMR, age-standardized mortality rate.

**Figure 4:**
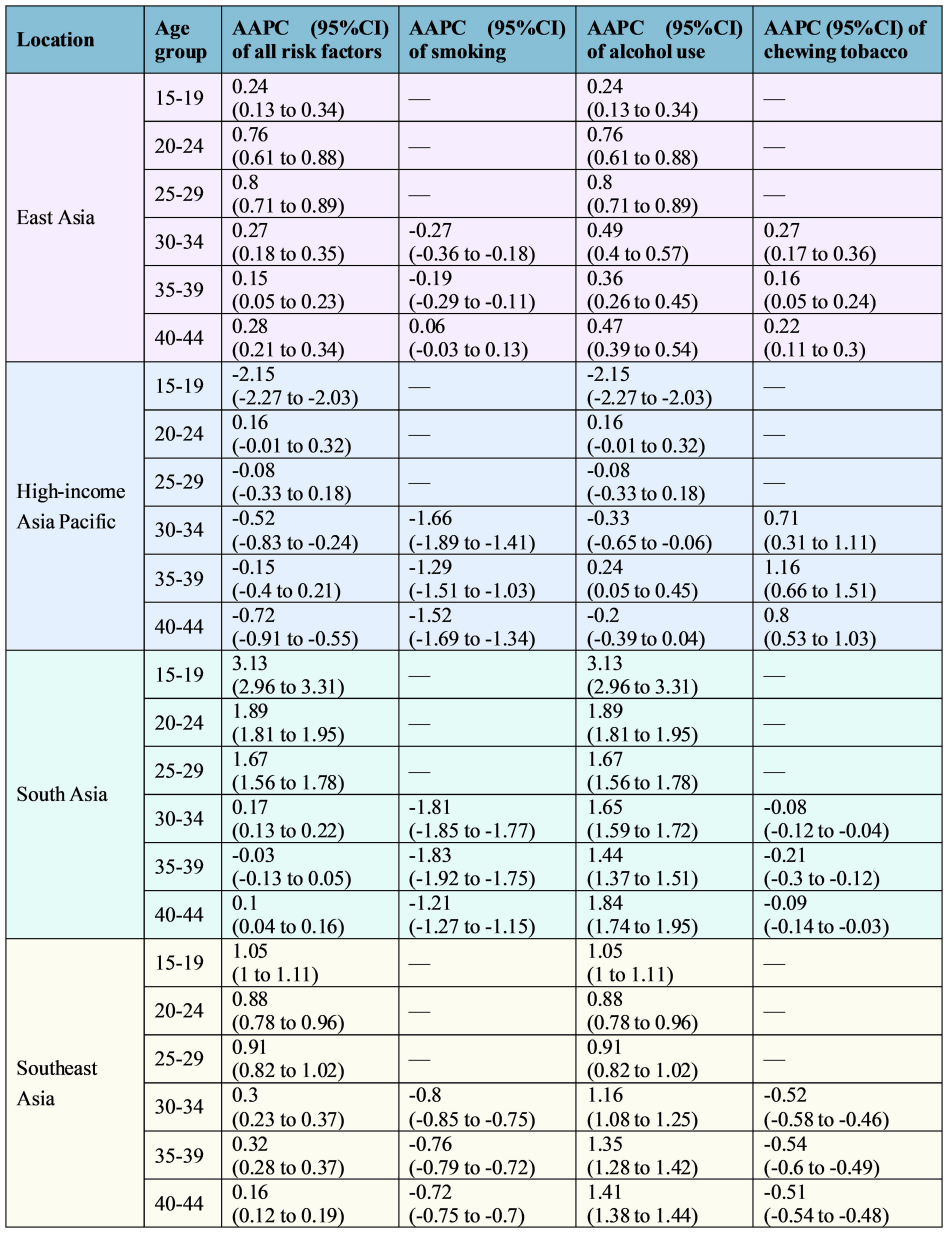
AAPC of lip and oral cavity cancer age-standardized DALYs rate attributable to each risk factor among young people (1990–2021), by age group. AAPC, average annual percent change; DALYs, disability-adjusted life-years.

Stratified by age group, from 1990 to 2021, the contributions of all risk factors to LOC deaths and DALYs among young populations increased in most age groups. This increase was most pronounced in the 25–29 age group in East Asia, and in the 15–19 age group in South Asia and Southeast Asia (**Figures 3**, **4**). Regarding smoking, its contribution showed a decreasing trend across most age groups in the four subregions. In contrast, the contributions of alcohol use demonstrated an increasing trend in all age subgroups in East Asia, South Asia, and Southeast Asia. The largest increases were observed in the 25–29 age subgroup in East Asia, the 15–19 age group in South Asia, and the 40–44 age group in Southeast Asia. As for chewing tobacco, its contribution increased across all age groups in East Asia and the High-income Asia Pacific, but decreased in South Asia and Southeast Asia.

### 3.5 Prediction to 2030

**Figure 5:**
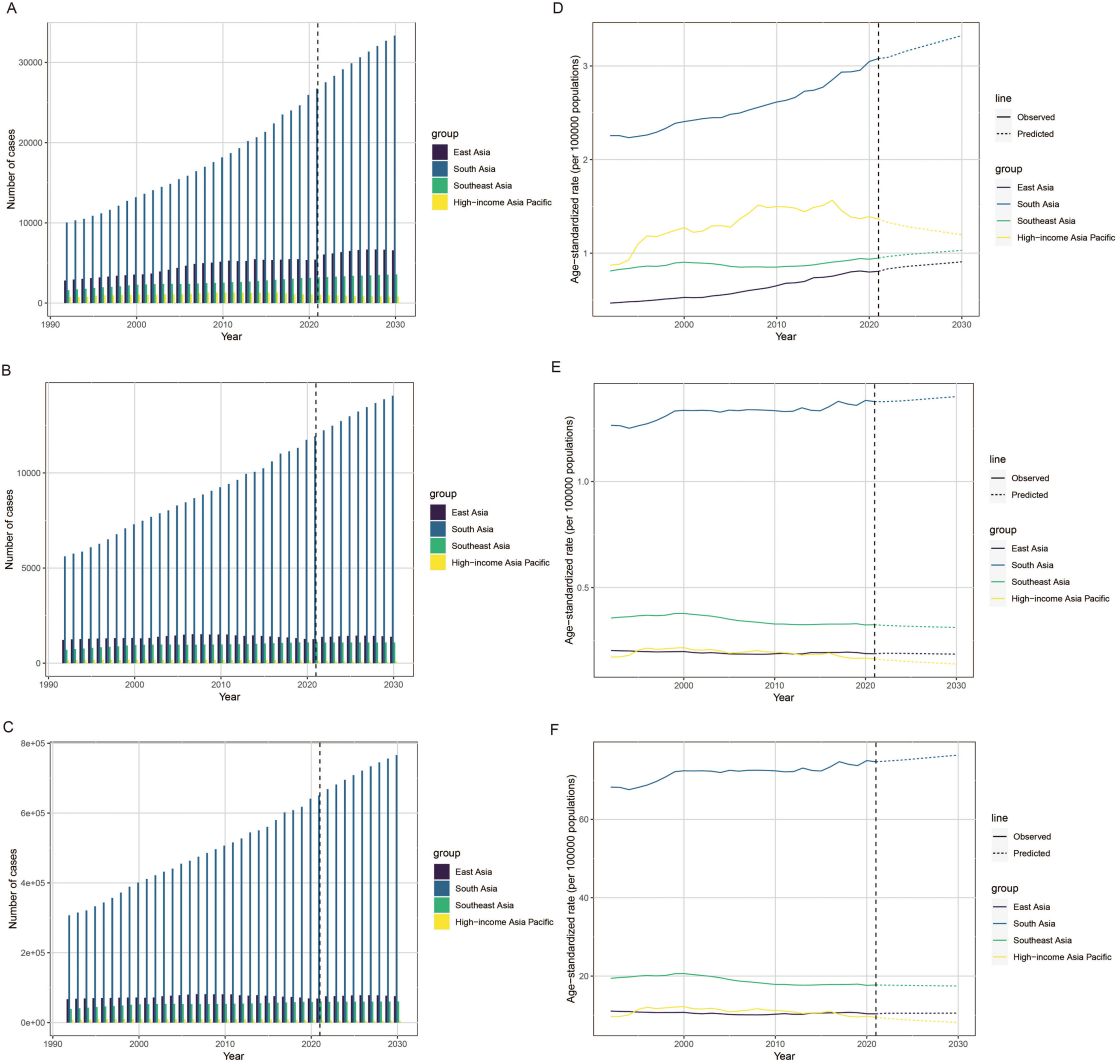
The projected global case numbers and ASR of lip and oral cavity cancer among young people to 2030. (1) The predicted case number of incidence **(A)**, deaths **(B)**, and DALYs **(C)**; (2) The predicted ASR of incidence **(D)**, deaths **(E)**, and DALYs **(F)**. ASR, age-standardized rate; DALYs, disability-adjusted life-years.

Nordpred models projected rising LOC incidence, deaths, and DALYs among young people in East, South, and Southeast Asia from 2022 to 2030, with South Asia showing the greatest increase (**Figure 5**, **Supplementary Table 9**). In contrast, the High-income Asia Pacific was predicted to exhibit consistent declines across all three indicators. As for the ASR, the ASIR was predicted to increase by 9.00% in East Asia, 7.54% in South Asia, and 6.87% in Southeast Asia, while declining 9.93% in High-income Asia Pacific. The ASMR was predicted to decrease annually in most subregions, except South Asia. Similarly, the age-standardized DALYs rate was projected to increase in East Asia and South Asia, while decreasing in High-income Asia Pacific and Southeast Asia. Sensitivity analyses using the lower and upper bounds of the data showed similar predicted trends, supporting the reliability of the projections (**Supplementary Tables 10**, **11**). Furthermore, residual analysis indicated an excellent model fit, with average relative errors well within the 10.00% threshold (**Supplementary Table 12**).

## 4 Discussion

South Asia remains the clear epicenter of early-onset LOC among young people (15–44 years) within the Asian subregions studied, with India and Pakistan as key hotspots. This aligns with Lin et al.’s findings, which identified South Asia as having the highest age-standardized DALY rate for LOC across all age groups in Asia (8). However, our age-stratified analysis reveals that the region’s high burden is driven by a distinct, adverse trend among young people: concurrent increases in the ASIR, ASMR, and age-standardized DALY rates—a pattern not observed in other subregions and not discernible in the all-age data. This escalation is particularly pronounced in the 20–24 age group. Furthermore, our application of the Nordpred model predicts a continued rise in these rates through 2030, offering new insights into the future trajectory of the epidemic. These findings indicate that the public health crisis in South Asia is not static but evolving, with a growing burden on young people that calls for targeted interventions.

Among Asian youth, the burden of LOC is heavily concentrated in South Asia—the only subregion exhibiting concurrent increases in ASIR, ASMR, and age-standardized DALYs rate. A global multicenter study supports this, reporting that Manipal, India, had the highest proportion of young oral squamous cell carcinoma (OSCC) patients (13.2%) among all institutions surveyed, highlighting the regional burden (18). Data from India’s National Cancer Registry Programme (NCRP) reaffirm that mouth and tongue cancer is a leading malignancy among adolescents and young adults (30–39 years), underscoring the persistent disease burden (19). India accounted for the highest case numbers of LOC incidence, deaths, and DALYs, attributable to its large population and the widespread consumption of smokeless tobacco (SLT) products, including khaini, gutka, and mawa (20). Our findings further validate chewing tobacco (a major SLT type) as the predominant risk factor for LOC deaths and DALYs among youth in this subregion. Despite a national gutka ban in 2011, illicit sales persist, sustaining high SLT use among youth (21). Regional disparities are marked, with states like Meghalaya and Gujarat showing extreme rates (22). In Pakistan, naswar—a widely used SLT product—accounts for nearly 70% of LOC cases in Khyber Pakhtunkhwa (23). This is supported by a recent hospital-based case-control study, which showed that naswar use significantly increases the risk of oral cancer (AOR = 6.83) (24). Compounding SLT’s impact, delayed diagnosis due to strained healthcare and limited public awareness worsens outcomes, while genetic vulnerabilities (GSTM1 and/or GSTT1 null genotypes and CYP1A1 variants) and HPV co-infection further amplify risks (25, 26).

Conversely, East and Southeast Asia demonstrate divergent epidemiological trends where rising ASIR coexist with declining ASMR and age-standardized DALYs rate. This pattern reflects distinct risk factor profiles and superior healthcare capacity. Particularly concerning is East Asia’s steep ASIR increase from 1990–2021, primarily driven by smoking (per GBD models) and emerging areca nut use—the latter being a major risk factor absent from GBD’s traditional framework. In Hunan, China, youth increasingly chew dried areca husk and swallow the juice—a high-risk behavior (27). In Taiwan (Province of China), youth experienced the steepest global increases in ASIR, ASMR, and age-standardized DALYs rate, despite declining areca nut use. This likely reflects legacy effects, as past chewers still face high risks (up to 137-fold for oral preneoplastic disorders) due to the long latency of oral carcinogenesis (27). Overall, the distribution of LOC in Asian youth closely follows regional patterns of SLT, areca nut, alcohol, and smoking, shaped by healthcare disparities and exacerbated by gene–virus interactions. Early, targeted interventions are urgently needed to curb this preventable burden.

From 1990 to 2021, South Asia showed the most pronounced early-onset LOC increase, particularly in 20–24-year-olds, while the proportion of non-smoking, non-drinking among OSCC cases continued to increase (28). These trends challenge traditional models of carcinogenesis, which assume prolonged exposure to smoking or alcohol. The risk profile of LOC is complex, with distinct regional patterns. While tobacco control has effectively reduced smoking-related burdens, the peak age-group attribution to alcohol varied by subregion (East Asia: 25–29; South Asia: 15–19; Southeast Asia: 40–44), and trends for chewing tobacco diverged (decreasing in South/Southeast Asia but increasing elsewhere). Alongside traditional factors, SLT and areca nut are established key causative agents. In 2022, they accounted for 31% of global LOC cases, primarily in lower-middle-income countries (29). Some products, like naswar, can increase LOC risk over 11-fold, though youth-specific data are scarce (30). Beyond these exogenous risk factors, emerging evidence highlights the role of biological mechanisms, particularly in early-onset and non-habit-related cases. First, HPV is an important causative factor of LOC. A recent systematic review across 11 Asian countries found the highest pooled HPV prevalence in OSCC in South Asia (27.1%), followed by East Asia (19.4%) and Southeast Asia (16.7%). Two subtypes—HPV-16 (9.1%) and HPV-18 (5.1%)—were most common (31). The E6 and E7 genes of high-risk HPV types can integrate into the host genome, disrupting cell proliferation and apoptosis through p53 degradation, retinoblastoma protein binding, and other mechanisms, promoting carcinogenesis (32). Second, genetic susceptibility is crucial. CASP8 mutations linked to impaired apoptosis (33), display widespread CpG island methylation (34), or carry undiagnosed Fanconi anemia (a condition conferring a 500-fold increased LOC risk) (35). Additionally, environmental exposure to tobacco smoke affects young women and is associated with tongue cancers (36). Microbial dysbiosis—marked by increased *Fusobacterium*, *Prevotella*, and *Alloprevotella* in young non-smokers—may drive inflammation, though causality is unclear (37). The coexistence of these diverse risk factors—ranging from substance use to infections and genetic predispositions—requires a multifaceted public health approach. In regions like South and Southeast Asia, where SLT and areca nut are major drivers, enforcing existing product bans is crucial. Globally, there is a pressing need to include areca nut control in cancer prevention policies. At the clinical level, screening protocols should be refined, such as prioritizing FANCA mutation testing in young, non-smoking, non-drinking patients to identify high-risk individuals early.

Globally, while young males exhibit a persistent male predominance in LOC incidence, mortality, and DALYs, females experienced significantly accelerated growth rates from 1990 to 2021 among young people (ASIR: +1.19% vs +0.67%; ASMR: +0.55% vs +0.21%; age-standardized DALYs rate: +0.59% vs +0.25%). Across Asian subregions, however, sex-specific trajectories diverge markedly: South Asia and High-income Asia Pacific mirror this global acceleration with female AAPC exceeding males (ASIR: female +1.13% vs male +1.05% in South Asia; female +1.89% vs male +1.32% in High-income Asia Pacific). Notably, East Asia exhibits the most extreme disparity, where male ASRs are over three times higher than those of females—largely attributable to higher rates of smoking and alcohol use among men (38). This shifting epidemiology within Asia finds parallels in independent global reports. A study of 89,212 tongue squamous cell carcinoma cases across 22 registries worldwide found a higher incidence increase among those under 45 in most regions, with a significant sex-based disparity in half of the registries. This suggests a broader shift toward younger individuals and women (39). Notably, analyses of cancer age distributions have identified oral cavity cancer as the only subsite showing a true increase in the proportion of young patients (0–39 years), strongly linked to rising incidence among young women (40). These external observations provide critical context and support for our region-specific findings, indicating that the rising burden among young women in parts of Asia is part of an emerging, though not universal, global pattern. The exacerbation of this burden in South Asia and high-income Asia-Pacific regions highlights a convergence of biological and structural vulnerabilities, which may be amplified in specific settings. First, hormonal dualism: estrogen can both suppress inflammation and promote tumor growth via ER-α overexpression, which is linked to worse prognosis in young women (41, 42). Second, women’s higher susceptibility to autoimmune disorders may compromise antitumor immunity (43). Third, HPV–sex interactions: although HPV-positive OSCC is more common in men, HPV-negative tumors—which are more frequent in women—tend to have poorer outcomes, suggesting intrinsic vulnerability (44, 45). In addition, diagnostic disparities may compound risk. These findings highlight the urgent need for sex-specific strategies, including routine screening for women regardless of behavioral history, targeted public health messaging on non-traditional exposures, and expanded research into sex-related biological mechanisms.

Nordpred projections show that LOC burden among youth (15–44 years) will rise across Asia from 2022 to 2030, with South Asia facing the steepest increases in incidence, mortality, and DALYs. In contrast, High-income Asia Pacific is expected to see steady declines, reflecting stronger health systems and preventive strategies. Population growth remains a key driver of global LOC burden and is projected to continue contributing to rising cases (46). According to the United Nations’ medium-variant projection (47), South Asia’s youth population (15–44 years) will grow by 7.18% and Southeast Asia’s by 3.51% by 2030, while East Asia’s will decline by 4.69%, potentially curbing its overall case growth. In resource-limited settings, the rising burden is exacerbated by weak screening systems. Although conventional visual and tactile examination (CVTE) is fast, affordable, and effective, screening rates remain under 40% (48), slowed by poor access and provider shortages. This hampers progress toward early detection goals outlined in the Global Strategy and Action Plan on Oral Health 2023–2030 (49), which emphasizes prevention, governance, improved data systems, and research. CVTE is especially valuable in high-incidence regions such as South Asia, where the risk posed by missed cases exceeds that of false positives. Meanwhile, HPV-driven LOC remains largely preventable, but many Asian countries have yet to integrate the HPV vaccine into national immunization programs (50). Barriers such as cost, logistics, regulation, and cultural resistance continue to limit uptake, especially among youth. High-income Asia Pacific’s declining burden likely stems from effective strategies: routine CVTE as part of dental care, supportive billing practices, and strong HPV vaccination programs. South Asia must adopt similar models, as its rising youth population, weak screening, and limited vaccine infrastructure create a perfect storm for a rising burden.

This study has several limitations. First, the accuracy of our estimates depends on the completeness and quality of the underlying source data. Biases may arise from underdiagnosis and variations in cancer registry coverage, especially in resource-limited settings. While the GBD’s modeling approach cannot replace local validation, the strong concordance of our core findings (e.g., regional burden rankings) with the GLOBOCAN database increases confidence that these macro-geographical patterns reflect a robust epidemiological reality. Second, the exclusion of areca nut as a distinct risk factor in GBD modeling limits the precise quantification of its burden in high-consumption regions. Third, the lack of data on anatomical sub-sites restricts deeper etiological analysis. Despite these limitations, our analysis offers a crucial macro-epidemiological perspective. Future research that integrates these high-level trends with localized primary data will be invaluable for validating and contextualizing our findings.

## 5 Conclusion

This study reveals three critical patterns in early-onset LOC burden among young people (15–44 years) across Asia: 1) South, East, and Southeast Asia carry persistently high burdens with steadily increasing ASIR from 1990 to 2021; 2) Projections indicate continued ASIR growth through 2030; and 3) South Asia exhibits the most concerning trajectory, with concurrent rises in ASIR, ASMR, and age-standardized DALYs rate—particularly in the 20–24 age group—while other subregions show declining mortality and DALYs rates. Within this landscape, India and Pakistan remain key hotspots. Addressing this escalating crisis demands urgent, subregion-specific interventions tailored to dominant risk factors. Simultaneously, the global health community must coordinate action through expanded research on early-onset aetiology, stronger international regulation of tobacco and alcohol targeting youth, and increased investment in surveillance and screening infrastructure.

## Data Availability

Publicly available datasets were analyzed in this study. This data can be found here: https://vizhub.healthdata.org/gbd-results/.
