# Supplementary material for: Burden of lip and oral cavity cancer among young people across South, East, and Southeast Asia: trends from 1990 to 2021 and predictions to 2030
Source: Front Oncol. 2026 Jan 16;15:1680008. doi: 10.3389/fonc.2025.1680008 (PMC12855057; doi:10.3389/fonc.2025.1680008)
Supplement: Supplementary file 1 [file DataSheet1.docx]

**Burden of lip and oral cavity cancer among young people across South, East, and Southeast Asia: trends from 1990 to 2021 and predictions to 2030**

**Table of contents**

[Supplementary Methods. 1](#_Toc219105222)

[Supplementary Table 1. 2](#_Toc219105223)

[Supplementary Table 2. 3](#_Toc219105224)

[Supplementary Table 3 5](#_Toc219105225)

[Supplementary Table 4. 7](#_Toc219105226)

[Supplementary Table 5 9](#_Toc219105227)

[Supplementary Table 6 12](#_Toc219105228)

[Supplementary Table 7 15](#_Toc219105229)

[Supplementary Table 8 18](#_Toc219105230)

[Supplementary Table 9 21](#_Toc219105231)

[Supplementary Table 10 26](#_Toc219105232)

[Supplementary Table 11 35](#_Toc219105233)

[Supplementary Table 12 44](#_Toc219105234)

Supplementary Methods. Principles of the Monte Carlo permutation test for determining the optimal number of joinpoints.

The optimal number of joinpoints in the Joinpoint regression model was determined using the Monte Carlo permutation test. Specifically, according to the principle of the Monte Carlo permutation test, we initially define the range for the number of joinpoints $k$ as $k\in(MIN, MAX)$, where MIN denotes the minimum number of joinpoints (typically set to 0) and MAX denotes the maximum number of joinpoints (set to 5 based on the data points). Each permutation test evaluates the null hypothesis $H_{0}$ (number of joinpoints $k=k_{a}$) against the alternative hypothesis $H_{1}$(number of joinpoints $k=k_{b}$). The permutation test starts with $k_{a}=MIN$ and $k_{b}=MAX$. If $H_{0}$ is rejected, we set $k=k_{a}+1$ and conduct the test again; if $H_{0}$ is not rejected, we set $k=k_{b}-1$ and conduct the test again. This process continues until $k_{a}=k_{b}$. At this point, the number of joinpoints ${k=k}_{a}=k_{b}$ is identified as the optimal number of joinpoints selected by the permutation test, and the corresponding model is the optimal model.

Supplementary Table 1. Complete list of countries within each subregion according to GBD 2021 regional classification standards.

| **Regions** | **Countries** |
| --- | --- |
| East Asia | China |
|  | Taiwan (Province of China) |
|  | Democratic People’s Republic of Korea |
| High-income Asia Pacific | Brunei Darussalam |
|  | Japan |
|  | Republic of Korea |
|  | Singapore |
| South Asia | Bangladesh |
|  | Bhutan |
|  | India |
|  | Nepal |
|  | Pakistan |
| Southeast Asia | Cambodia |
|  | Indonesia |
|  | Lao People’s Democratic Republic |
|  | Malaysia |
|  | Maldives |
|  | Mauritius |
|  | Myanmar |
|  | Philippines |
|  | Seychelles |
|  | Sri Lanka |
|  | Thailand |
|  | Timor-Leste |
|  | Viet Nam |

GBD: Global Burden of Disease.

Supplementary Table 2. Joinpoint regression residual analysis of ASIR, ASMR, and age-standardized DALYs rate for lip and oral cavity cancer, 1990-2021.

| **Location** | **Average relative error (%)** | | |
| --- | --- | --- | --- |
|  | **ASIR** | **ASMR** | **Age-standardized DALYs rate** |
| Global | 0.1911 | 0.3259 | 0.2119 |
| **Regions** | | | |
| East Asia | 0.7312 | 0.1562 | 0.565 |
| High-income Asia Pacific | 1.4847 | 2.5017 | 1.852 |
| South Asia | 0.4137 | 0.3532 | 0.3564 |
| Southeast Asia | 0.5005 | 0.3717 | 0.4064 |
| **Countries** | | | |
| Bangladesh | 0.6415 | 0.501 | 0.5847 |
| Bhutan | 0.3075 | 0.5053 | 0.5595 |
| Brunei Darussalam | 0.5814 | 0.6599 | 0.6534 |
| Cambodia | 0.2175 | 0.4102 | 0.2464 |
| China | 1.0492 | 0.5551 | 0.6468 |
| Democratic People's Republic of Korea | 0.1774 | 0.1562 | 0.1246 |
| India | 0.3744 | 0.5742 | 0.6019 |
| Indonesia | 0.3908 | 0 | 0.1033 |
| Japan | 1.9269 | 2.6437 | 2.5261 |
| Lao People's Democratic Republic | 0.1851 | 0.2158 | 0.2983 |
| Malaysia | 1.5125 | 2.0894 | 1.8226 |
| Maldives | 1.1873 | 1.9792 | 1.9869 |
| Mauritius | 2.7113 | 3.2848 | 2.9856 |
| Myanmar | 0.4292 | 0.125 | 0.2611 |
| Nepal | 0.271 | 0.1832 | 0.1984 |
| Pakistan | 0.3318 | 0.2209 | 0.238 |
| Philippines | 0.0933 | 0.3154 | 0.5965 |
| Republic of Korea | 0.5929 | 1.242 | 0.7366 |
| Seychelles | 2.464 | 2.6202 | 2.5546 |
| Singapore | 4.8375 | 5.6017 | 5.8534 |
| Sri Lanka | 1.5378 | 1.5392 | 1.3077 |
| Taiwan (Province of China) | 1.9883 | 2.0775 | 2.0084 |
| Thailand | 1.3195 | 1.4693 | 1.3459 |
| Timor-Leste | 1.1491 | 1.1722 | 1.5704 |
| Viet Nam | 0.1688 | 0.0762 | 0.1866 |

ASIR: age-standardized incidence rate; ASMR: age-standardized mortality rate; DALYs: disability-adjusted life-years.

Supplementary Table 3**.** Sensitivity analysis of Joinpoint models using lower/upper bounds of ASIR, ASMR, and age-standardized DALYs rate for lip and oral cavity cancer, 1990-2021.

| **Location** | **ASIR** | | | **ASMR** | | | **age-standardized DALYs rate** | | |
| --- | --- | --- | --- | --- | --- | --- | --- | --- | --- |
|  | **Original AAPC (95% CI)** | **Lower Bound AAPC (95%CI)** | **Upper Bound AAPC (95%CI)** | **Original AAPC (95% CI)** | **Lower Bound AAPC (95%CI)** | **Upper Bound AAPC (95%CI)** | **Original AAPC (95% CI)** | **Lower Bound-AAPC (95%CI)** | **Upper Bound -AAPC (95%CI)** |
| Global | 0.81 (0.75 to 0.85) | 0.53 (0.48 to 0.58) | 0.99 (0.95 to 1.02) | 0.34 (0.31 to 0.36) | 0.01 (-0.04 to 0.06) | 0.47 (0.44 to 0.5) | 0.37 (0.34 to 0.4) | 0.02 (-0.02 to 0.06) | 0.52 (0.5 to 0.55) |
| **Regions** |  |  |  |  |  |  |  |  |  |
| East Asia | 1.84 (1.75 to 1.92) | 1.74 (1.67 to 1.81) | 1.99 (1.9 to 2.07) | -0.31 (-0.41 to -0.22) | -0.36 (-0.46 to -0.26) | -0.13 (-0.24 to -0.05) | -0.26 (-0.37 to -0.18) | -0.42 (-0.54 to -0.28) | -0.1 (-0.2 to -0.02) |
| High-income Asia Pacific | 1.47 (1.24 to 1.63) | 1.32 (1.13 to 1.49) | 1.61 (1.43 to 1.77) | -0.19 (-0.52 to 0.12) | -0.24 (-0.49 to 0.02) | -0.03 (-0.2 to 0.19) | -0.1 (-0.48 to 0.24) | -0.15 (-0.42 to 0.11) | 0.08 (-0.09 to 0.3) |
| South Asia | 1.06 (1.02 to 1.1) | 0.77 (0.71 to 0.83) | 1.24 (1.17 to 1.31) | 0.33 (0.29 to 0.37) | 0 (-0.06 to 0.07) | 0.47 (0.43 to 0.51) | 0.35 (0.31 to 0.39) | 0.01 (-0.06 to 0.07) | 0.51 (0.47 to 0.55) |
| Southeast Asia | 0.63 (0.58 to 0.68) | 0.61 (0.55 to 0.66) | 0.69 (0.65 to 0.72) | -0.24 (-0.27 to -0.2) | -0.28 (-0.32 to -0.24) | -0.18 (-0.22 to -0.15) | -0.24 (-0.27 to -0.2) | -0.25 (-0.29 to -0.22) | -0.19 (-0.22 to -0.16) |
| **Countries** |  |  |  |  |  |  |  |  |  |
| Bangladesh | 0.43 (0.37 to 0.48) | -0.33 (-0.45 to -0.25) | 0.74 (0.69 to 0.78) | -0.77 (-0.83 to -0.73) | -1.55 (-1.64 to -1.47) | -0.38 (-0.45 to -0.33) | -0.7 (-0.76 to -0.66) | -1.45 (-1.55 to -1.37) | -0.31 (-0.38 to -0.26) |
| Bhutan | 0.45 (0.39 to 0.54) | 0.33 (0.24 to 0.43) | 0.81 (0.74 to 0.89) | -0.56 (-0.61 to -0.51) | -0.8 (-0.9 to -0.69) | -0.28 (-0.35 to -0.21) | -0.52 (-0.57 to -0.46) | -0.77 (-0.87 to -0.66) | -0.24 (-0.31 to -0.18) |
| Brunei Darussalam | 0.08 (-0.02 to 0.18) | 0.11 (0.03 to 0.19) | -0.02 (-0.13 to 0.1) | -0.87 (-0.96 to -0.77) | -0.78 (-0.86 to -0.71) | -0.95 (-1.06 to -0.83) | -0.86 (-0.97 to -0.74) | -0.76 (-0.84 to -0.68) | -0.98 (-1.09 to -0.86) |
| Cambodia | 1.12 (1.1 to 1.14) | 1.28 (1.25 to 1.32) | 1.06 (1.01 to 1.1) | 0.34 (0.32 to 0.37) | 0.49 (0.45 to 0.53) | 0.34 (0.29 to 0.38) | 0.35 (0.32 to 0.37) | 0.5 (0.46 to 0.54) | 0.34 (0.29 to 0.38) |
| China | 1.74 (1.6 to 1.81) | 1.55 (1.46 to 1.64) | 1.86 (1.75 to 1.94) | -0.58 (-0.69 to -0.51) | -0.8 (-0.96 to -0.65) | -0.39 (-0.48 to -0.32) | -0.52 (-0.64 to -0.44) | -0.66 (-0.75 to -0.59) | -0.33 (-0.43 to -0.26) |
| Democratic People's Republic of Korea | 0.36 (0.33 to 0.38) | 0.36 (0.32 to 0.41) | 0.45 (0.4 to 0.5) | -0.26 (-0.27 to -0.25) | -0.28 (-0.32 to -0.24) | -0.14 (-0.18 to -0.11) | -0.25 (-0.26 to -0.24) | -0.28 (-0.32 to -0.24) | -0.15 (-0.19 to -0.12) |
| India | 0.89 (0.83 to 0.94) | 0.55 (0.44 to 0.64) | 1.08 (1.02 to 1.13) | 0.05 (0 to 0.1) | -0.27 (-0.37 to -0.19) | 0.22 (0.16 to 0.27) | 0.05 (0 to 0.09) | -0.29 (-0.38 to -0.21) | 0.22 (0.16 to 0.28) |
| Indonesia | 0.45 (0.43 to 0.47) | 0.44 (0.39 to 0.5) | 0.58 (0.54 to 0.62) | -0.19 (-0.2 to -0.17) | -0.25 (-0.3 to -0.2) | 0.02 (-0.02 to 0.07) | -0.18 (-0.19 to -0.16) | -0.22 (-0.27 to -0.18) | 0.02 (-0.02 to 0.08) |
| Japan | 1.55 (1.08 to 1.95) | 1.42 (1.06 to 1.83) | 1.7 (1.46 to 1.9) | 0.28 (-0.06 to 0.56) | 0.28 (-0.01 to 0.55) | 0.31 (0 to 0.59) | 0.39 (0.07 to 0.66) | 0.38 (0.1 to 0.66) | 0.43 (0.14 to 0.71) |
| Lao People's Democratic Republic | 0.39 (0.37 to 0.42) | 0.52 (0.49 to 0.54) | 0.05 (0.01 to 0.09) | -0.37 (-0.39 to -0.34) | -0.31 (-0.34 to -0.26) | -0.67 (-0.71 to -0.63) | -0.33 (-0.35 to -0.3) | -0.27 (-0.31 to -0.23) | -0.63 (-0.67 to -0.59) |
| Malaysia | 0.75 (0.64 to 0.86) | 0.79 (0.66 to 0.9) | 0.7 (0.59 to 0.82) | -0.29 (-0.43 to -0.16) | -0.22 (-0.36 to -0.1) | -0.28 (-0.42 to -0.15) | -0.29 (-0.44 to -0.16) | -0.21 (-0.35 to -0.09) | -0.29 (-0.43 to -0.16) |
| Maldives | 0.1 (-0.1 to 0.28) | 1.36 (1.22 to 1.47) | -0.73 (-0.94 to -0.53) | -1.31 (-1.59 to -1.05) | -0.3 (-0.4 to -0.21) | -2.23 (-2.43 to -2.02) | -1.28 (-1.53 to -1.02) | -0.26 (-0.36 to -0.17) | -2.17 (-2.38 to -1.96) |
| Mauritius | 0.85 (0.54 to 1.17) | 0.6 (0.25 to 1) | 0.93 (0.63 to 1.25) | 0.33 (-0.02 to 0.75) | 0.08 (-0.23 to 0.57) | 0.3 (-0.22 to 0.64) | 0.12 (-0.19 to 0.59) | 0.11 (-0.2 to 0.6) | 0.32 (-0.19 to 0.66) |
| Myanmar | 0.05 (0.02 to 0.08) | 0.48 (0.44 to 0.51) | -0.27 (-0.38 to -0.16) | -0.76 (-0.78 to -0.74) | -0.35 (-0.4 to -0.31) | -1.16 (-1.22 to -1.11) | -0.75 (-0.77 to -0.72) | -0.32 (-0.37 to -0.28) | -1.14 (-1.2 to -1.09) |
| Nepal | 0.82 (0.79 to 0.84) | 0.68 (0.65 to 0.72) | 0.86 (0.79 to 0.93) | -0.07 (-0.09 to -0.05) | -0.24 (-0.3 to -0.18) | -0.13 (-0.18 to -0.09) | -0.05 (-0.08 to -0.03) | -0.24 (-0.3 to -0.18) | -0.1 (-0.15 to -0.05) |
| Pakistan | 1.19 (1.15 to 1.22) | 1.02 (0.96 to 1.08) | 1.31 (1.24 to 1.39) | 0.75 (0.72 to 0.78) | 0.55 (0.51 to 0.6) | 0.89 (0.83 to 0.95) | 0.79 (0.76 to 0.81) | 0.59 (0.55 to 0.64) | 0.93 (0.87 to 0.98) |
| Philippines | -0.6 (-0.64 to -0.55) | -0.62 (-0.68 to -0.55) | -0.47 (-0.59 to -0.4) | -0.84 (-0.9 to -0.79) | -0.91 (-0.99 to -0.85) | -0.79 (-0.96 to -0.69) | -0.85 (-0.91 to -0.79) | -0.91 (-0.99 to -0.86) | -0.79 (-0.97 to -0.69) |
| Republic of Korea | 1.84 (1.77 to 1.92) | 1.49 (1.42 to 1.58) | 2.1 (2.02 to 2.19) | -1.15 (-1.23 to -1.07) | -1.42 (-1.5 to -1.36) | -0.85 (-0.93 to -0.78) | -1.02 (-1.12 to -0.93) | -1.3 (-1.38 to -1.22) | -0.74 (-0.82 to -0.66) |
| Seychelles | 1.21 (0.96 to 1.44) | 1 (0.71 to 1.28) | 1.23 (0.83 to 1.52) | 0.54 (0.26 to 0.79) | 0.51 (0.24 to 0.8) | 0.48 (0.09 to 0.76) | 0.57 (0.28 to 0.83) | 0.49 (0.21 to 0.8) | 0.52 (0.11 to 0.83) |
| Singapore | 0.63 (0.07 to 1.05) | 0.49 (-0.06 to 0.94) | 0.54 (0 to 0.99) | -1.42 (-1.82 to -0.79) | -1.75 (-2.31 to -1.28) | -1.69 (-2.27 to -1.2) | -1.19 (-1.6 to -0.58) | -1.25 (-1.64 to -0.66) | -1.18 (-1.58 to -0.59) |
| Sri Lanka | 1.13 (0.82 to 1.35) | 0.54 (0.24 to 0.79) | 1.65 (1.33 to 1.92) | 0.01 (-0.29 to 0.23) | -0.71 (-1.01 to -0.45) | 0.58 (0.33 to 0.79) | -0.06 (-0.39 to 0.18) | -0.75 (-1.08 to -0.45) | 0.52 (0.28 to 0.74) |
| Taiwan (Province of China) | 2.87 (2.71 to 3.03) | 2.67 (2.52 to 2.84) | 3.07 (2.91 to 3.24) | 1.67 (1.5 to 1.83) | 1.47 (1.29 to 1.63) | 1.77 (1.6 to 1.95) | 1.6 (1.43 to 1.77) | 1.48 (1.33 to 1.65) | 1.71 (1.54 to 1.89) |
| Thailand | 1.81 (1.71 to 1.91) | 1.6 (1.36 to 1.8) | 1.77 (1.69 to 1.86) | 0.54 (0.43 to 0.65) | 0.42 (0.16 to 0.6) | 0.49 (0.42 to 0.59) | 0.55 (0.45 to 0.67) | 0.47 (0.2 to 0.69) | 0.58 (0.5 to 0.66) |
| Timor-Leste | 0.78 (0.68 to 0.88) | 0.8 (0.6 to 0.99) | 0.82 (0.72 to 0.93) | 0.23 (0.12 to 0.33) | 0.29 (0.08 to 0.49) | 0.36 (0.26 to 0.46) | 0.23 (0.11 to 0.34) | 0.29 (0.07 to 0.5) | 0.35 (0.24 to 0.45) |
| Viet Nam | 1.12 (1.09 to 1.14) | 1.12 (1.06 to 1.18) | 1.24 (1.2 to 1.27) | -0.14 (-0.16 to -0.13) | -0.24 (-0.27 to -0.2) | 0.05 (0 to 0.1) | -0.11 (-0.12 to -0.09) | -0.2 (-0.24 to -0.16) | 0.06 (0.02 to 0.11) |

AAPC: average annual percent change, ASIR: age-standardized incidence rate, ASMR: age-standardized mortality rate, DALYs: disability-adjusted life-years.

Supplementary Table 4. Number of cases and ASR of lip and oral cavity incidence and deaths among young people in 1990 and 2021 (GBD 2021) and 2022 (GLOBOCAN 2022), by country in four Asian regions.

| **Countries** | **Incidence** | | | | **Deaths** | | | |
| --- | --- | --- | --- | --- | --- | --- | --- | --- |
|  | Number  in GBD 2021 | Number in GLOBOCAN 2022 | ASR  in GBD 2021 | ASR in GLOBOCAN 2022 | Number  in GBD 2021 | Number in GLOBOCAN 2022 | ASR  in GBD 2021 | ASR in GLOBOCAN 2022 |
| Bangladesh | 1539  (730-2597) | 2457 | 2.02  (0.96-3.4) | 2.8 | 625  (299-1075) | 1240 | 0.82  (0.39-1.41) | 1.4 |
| Bhutan | 8  (4-13) | 2 | 1.97  (1.03-3.4) | 0.46 | 3  (2-6) | 0 | 0.85  (0.43-1.46) | 0 |
| Brunei Darussalam | 4  (3-5) | 0 | 1.47  (1.02-2.04) | 0 | 1  (1-1) | 0 | 0.4  (0.29-0.55) | 0 |
| Cambodia | 57  (36-89) | 58 | 0.72  (0.46-1.13) | 0.68 | 25  (16-40) | 23 | 0.32  (0.2-0.51) | 0.27 |
| China | 4535  (3648-5574) | 2674 | 0.7  (0.57-0.86) | 0.39 | 1062  (852-1310) | 732 | 0.16  (0.13-0.2) | 0.1 |
| Democratic People's Republic of Korea | 74  (44-123) | 25 | 0.58  (0.34-0.97) | 0.21 | 26  (15-43) | 7 | 0.2  (0.12-0.33) | 0.06 |
| India | 17948  (14322-21224) | 29212 | 2.65  (2.12-3.13) | 4 | 7801  (6175-9252) | 14098 | 1.16  (0.92-1.37) | 2 |
| Indonesia | 832  (610-1124) | 641 | 0.59  (0.43-0.8) | 0.45 | 343  (248-464) | 242 | 0.24  (0.18-0.33) | 0.17 |
| Japan | 754  (639-895) | 450 | 1.59  (1.35-1.89) | 0.94 | 91  (88-94) | 69 | 0.19  (0.18-0.19) | 0.14 |
| Lao People's Democratic Republic | 20  (12-30) | 36 | 0.58  (0.35-0.89) | 1 | 9  (6-14) | 18 | 0.28  (0.17-0.42) | 0.51 |
| Malaysia | 187  (123-277) | 79 | 1.17  (0.77-1.73) | 0.45 | 57  (38-83) | 32 | 0.36  (0.24-0.52) | 0.18 |
| Maldives | 2  (1-3) | 0 | 0.58  (0.38-0.84) | 0 | 1  (0-1) | 0 | 0.16  (0.1-0.24) | 0 |
| Mauritius | 7  (5-8) | — | 1.07  (0.88-1.29) | — | 2  (2-2) | — | 0.35  (0.3-0.4) | — |
| Myanmar | 143  (86-221) | 370 | 0.56  (0.34-0.86) | 1.4 | 61  (37-94) | 174 | 0.24  (0.14-0.37) | 0.63 |
| Nepal | 260  (158-406) | 115 | 1.93  (1.18-3.01) | 0.85 | 116  (71-181) | 46 | 0.87  (0.53-1.35) | 0.33 |
| Pakistan | 6976  (4854-9707) | 3903 | 6.89  (4.82-9.54) | 3.8 | 3376  (2383-4610) | 2261 | 3.35  (2.38-4.56) | 2.2 |
| Philippines | 349  (288-426) | 162 | 0.68  (0.56-0.83) | 0.31 | 145  (121-171) | 58 | 0.28  (0.24-0.33) | 0.11 |
| Republic of Korea | 241  (164-346) | 121 | 1.01  (0.69-1.45) | 0.51 | 30  (22-42) | 27 | 0.12  (0.09-0.17) | 0.11 |
| Seychelles | 1  (1-2) | — | 2.7  (1.69-4.12) | — | 1  (0-1) | — | 1.01  (0.63-1.5) | — |
| Singapore | 24  (19-31) | 23 | 0.76  (0.59-0.97) | 0.77 | 3  (3-4) | 4 | 0.1  (0.08-0.12) | 0.13 |
| Sri Lanka | 184  (99-300) | 166 | 1.8  (0.97-2.91) | 1.6 | 53  (29-86) | 70 | 0.51  (0.28-0.83) | 0.69 |
| Taiwan  (Province of China) | 800  (622-1015) | — | 6.28  (4.88-7.96) | — | 173  (142-208) | — | 1.33  (1.09-1.6) | — |
| Thailand | 714  (473-1007) | 299 | 2.29  (1.52-3.24) | 0.92 | 192  (128-270) | 100 | 0.6  (0.4-0.85) | 0.3 |
| Timor-Leste | 2  (1-4) | 0 | 0.45  (0.27-0.71) | 0 | 1  (1-2) | 0 | 0.21  (0.13-0.34) | 0 |
| Viet Nam | 714  (465-1097) | 287 | 1.41  (0.92-2.16) | 0.56 | 215  (138-334) | 113 | 0.42  (0.27-0.66) | 0.22 |

ASR: age-standardized rate; GBD: Global Burden of Disease; GLOBOCAN: Global Cancer Observatory.

Supplementary Table 5**.** Number of cases and ASR of lip and oral cavity cancer incidence, deaths, and DALYs among young people (1990, 2021) across the four Asian regions, with AAPC from 1990 to 2021, by sex.

| **Metrics** | **Location** | **1990** | | **2021** | | **AAPC (95%CI)** | ***P*** |
| --- | --- | --- | --- | --- | --- | --- | --- |
|  |  | **Number (95%UI)** | **ASR (95%UI)** | **Number (95%UI)** | **ASR (95%UI)** |  |  |
| **Incidence** | **Global** | | | | | | |
|  | Male | 15415(14144-16619) | 1.37(1.26-1.48) | 30179(25273-34074) | 1.67(1.4-1.89) | 0.67  (0.63 to 0.71) | <0.001 |
|  | Female | 8410(7724-9200) | 0.75(0.69-0.82) | 19031(16286-22404) | 1.08(0.93-1.28) | 1.19  (1.14 to 1.22) | <0.001 |
|  | **East Asia** | | | | | | |
|  | Male | 1537(1276-1815) | 0.53(0.44-0.62) | 3933(3153-4901) | 1.13(0.9-1.41) | 2.49 (2.34 to 2.6) | <0.001 |
|  | Female | 1048(830-1312) | 0.38(0.3-0.47) | 1476(1108-1949) | 0.46(0.35-0.61) | 0.67 (0.61 to 0.74) | <0.001 |
|  | **High-income Asia Pacific** | | | | | | |
|  | Male | 429(375-494) | 0.99(0.86-1.14) | 583(472-706) | 1.49(1.21-1.81) | 1.32 (1.16 to 1.46) | <0.001 |
|  | Female | 286(238-343) | 0.68(0.57-0.82) | 439(352-539) | 1.23(0.98-1.51) | 1.89 (1.62 to 2.15) | <0.001 |
|  | **South Asia** | | | | | | |
|  | Male | 5662(4664-6704) | 2.6(2.15-3.08) | 15909(11888-19140) | 3.63(2.72-4.37) | 1.05 (0.97 to 1.13) | <0.001 |
|  | Female | 3653(3113-4259) | 1.77(1.51-2.06) | 10820(8343-13965) | 2.51(1.94-3.23) | 1.13 (1.03 to 1.2) | <0.001 |
|  | **Southeast Asia** | | | | | | |
|  | Male | 781(635-954) | 0.87(0.7-1.06) | 1894(1483-2365) | 1.1(0.86-1.38) | 0.79 (0.74 to 0.84) | <0.001 |
|  | Female | 669(534-820) | 0.7(0.56-0.85) | 1323(1058-1644) | 0.79(0.63-0.98) | 0.41 (0.37 to 0.44) | <0.001 |
| **Deaths** | **Global** | | | | | | |
|  | Male | 6826(6118-7535) | 0.61(0.55-0.67) | 11724(9523-13480) | 0.65(0.53-0.75) | 0.21(0.18 to 0.25) | <0.001 |
|  | Female | 3335(3014-3720) | 0.3(0.27-0.33) | 6155(5131-7489) | 0.35(0.29-0.43) | 0.55(0.46 to 0.6) | <0.001 |
|  | **East Asia** | | | | | | |
|  | Male | 758(624-906) | 0.26(0.22-0.31) | 1017(823-1265) | 0.29(0.24-0.36) | 0.33 (0.22 to 0.41) | <0.001 |
|  | Female | 401(317-502) | 0.14(0.11-0.18) | 244(184-320) | 0.08(0.06-0.1) | -2.02 (-2.08 to -1.97) | <0.001 |
|  | **High-income Asia Pacific** | | | | | | |
|  | Male | 96(88-105) | 0.22(0.2-0.24) | 80(72-89) | 0.2(0.18-0.22) | -0.3 (-0.51 to -0.04) | 0.034 |
|  | Female | 50(46-54) | 0.12(0.11-0.13) | 46(42-51) | 0.12(0.11-0.14) | 0.17 (-0.17 to 0.45) | 0.284 |
|  | **South Asia** | | | | | | |
|  | Male | 3356(2769-3973) | 1.55(1.28-1.83) | 7605(5722-9192) | 1.74(1.31-2.1) | 0.36 (0.29 to 0.42) | <0.001 |
|  | Female | 1882(1609-2194) | 0.92(0.78-1.07) | 4317(3365-5570) | 1(0.79-1.29) | 0.31 (0.2 to 0.4) | <0.001 |
|  | **Southeast Asia** | | | | | | |
|  | Male | 399(327-485) | 0.44(0.36-0.54) | 745(592-917) | 0.43(0.34-0.53) | -0.07 (-0.1 to -0.03) | <0.001 |
|  | Female | 248(198-307) | 0.26(0.21-0.32) | 360(295-440) | 0.21(0.18-0.26) | -0.61 (-0.63 to -0.58) | <0.001 |
| **DALYs** | **Global** | | | | | | |
|  | Male | 367910(329617-406845) | 32.44(29.1-35.81) | 630700(511034-725878) | 35.06(28.39-40.36) | 0.25(0.21 to 0.29) | <0.001 |
|  | Female | 187448(169036-209074) | 16.55(14.94-18.43) | 344928(285126-421936) | 19.73(16.28-24.17) | 0.59(0.51 to 0.65) | <0.001 |
|  | **East Asia** | | | | | | |
|  | Male | 41148(33829-49180) | 14.03(11.54-16.78) | 54895(44224-68092) | 15.89(12.78-19.73) | 0.43 (0.29 to 0.53) | <0.001 |
|  | Female | 22803(18076-28651) | 8.08(6.4-10.14) | 13828(10435-18214) | 4.44(3.36-5.83) | -1.93 (-1.98 to -1.87) | <0.001 |
|  | **High-income Asia Pacific** | | | | | | |
|  | Male | 5263(4832-5814) | 12.08(11.06-13.38) | 4468(4014-5012) | 11.41(10.24-12.82) | -0.21 (-0.41 to 0.04) | 0.086 |
|  | Female | 2793(2555-3085) | 6.67(6.08-7.39) | 2643(2402-2957) | 7.42(6.74-8.3) | 0.48 (0.11 to 0.82) | 0.03 |
|  | **South Asia** | | | | | | |
|  | Male | 181610(149653-215503) | 82.65(68.17-97.94) | 408560(305706-494926) | 93(69.74-112.55) | 0.36 (0.29 to 0.42) | <0.001 |
|  | Female | 105309(89887-122769) | 50.21(42.94-58.47) | 241909(186584-314963) | 55.92(43.29-72.6) | 0.36 (0.26 to 0.44) | <0.001 |
|  | **Southeast Asia** | | | | | | |
|  | Male | 21874(17950-26572) | 23.92(19.61-29.03) | 40066(31808-49218) | 23.4(18.58-28.74) | -0.06 (-0.1 to -0.03) | <0.001 |
|  | Female | 14079(11156-17440) | 14.47(11.55-17.82) | 19967(16323-24342) | 11.96(9.77-14.58) | -0.59 (-0.62 to -0.57) | <0.001 |

AAPC: average annual percent change; ASR: age-standardized rate; DALYs: age-standardized disability-adjusted life years.

Supplementary Table 6**.** Number of cases and ASR of lip and oral cavity cancer incidence among young people (1990, 2021), with AAPC from 1990 to 2021, by age group.

| **Location** | **Age group** | **1990** | | **2021** | | **AAPC (95%CI)** | ***P*** |
| --- | --- | --- | --- | --- | --- | --- | --- |
|  |  | **Number (95%UI)** | **ASR (95%UI)** | **Number (95%UI)** | **ASR (95%UI)** |  |  |
| **Global** | 15-19 | 765(705-842) | 0.15(0.14-0.16) | 1277(988-1559) | 0.2(0.16-0.25) | 1.09 (1 to 1.14) | <0.001 |
|  | 20-24 | 1190(1109-1283) | 0.24(0.23-0.26) | 2282(1801-2795) | 0.38(0.3-0.47) | 1.51 (1.43 to 1.57) | <0.001 |
|  | 25-29 | 1982(1832-2130) | 0.45(0.41-0.48) | 3986(3266-4646) | 0.68(0.56-0.79) | 1.38 (1.3 to 1.44) | <0.001 |
|  | 30-34 | 3464(3214-3707) | 0.9(0.83-0.96) | 7753(6602-8767) | 1.28(1.09-1.45) | 1.14 (1.1 to 1.19) | <0.001 |
|  | 35-39 | 6695(6249-7123) | 1.9(1.77-2.02) | 13924(12213-15424) | 2.48(2.18-2.75) | 0.85 (0.81 to 0.88) | <0.001 |
|  | 40-44 | 9729(9211-10263) | 3.4(3.22-3.58) | 19987(17761-21757) | 4(3.55-4.35) | 0.53 (0.48 to 0.58) | <0.001 |
| **East Asia** | 15-19 | 124(107-143) | 0.1(0.08-0.11) | 90(72-108) | 0.12(0.09-0.14) | 0.7 (0.63 to 0.76) | <0.001 |
|  | 20-24 | 158(135-185) | 0.12(0.1-0.14) | 153(127-183) | 0.2(0.17-0.24) | 1.76 (1.61 to 1.88) | <0.001 |
|  | 25-29 | 205(174-239) | 0.18(0.15-0.21) | 301(247-361) | 0.33(0.27-0.4) | 2.08 (1.94 to 2.22) | <0.001 |
|  | 30-34 | 421(365-485) | 0.46(0.4-0.53) | 1021(845-1213) | 0.82(0.68-0.97) | 1.81 (1.68 to 1.92) | <0.001 |
|  | 35-39 | 792(676-909) | 0.84(0.72-0.96) | 1662(1392-1958) | 1.51(1.27-1.78) | 1.9 (1.79 to 2) | <0.001 |
|  | 40-44 | 884(762-1016) | 1.27(1.1-1.46) | 2182(1807-2617) | 2.29(1.89-2.74) | 1.97 (1.87 to 2.06) | <0.001 |
| **High-income Asia Pacific** | 15-19 | 14(13-16) | 0.12(0.09-0.14) | 12(10-14) | 0.12(0.11-0.15) | 1.36 (1.17 to 1.54) | <0.001 |
|  | 20-24 | 32(28-36) | 0.21(0.17-0.26) | 41(35-47) | 0.23(0.19-0.27) | 1.98 (1.78 to 2.17) | <0.001 |
|  | 25-29 | 67(59-76) | 0.39(0.32-0.48) | 94(80-109) | 0.46(0.38-0.54) | 1.64 (1.31 to 1.97) | <0.001 |
|  | 30-34 | 118(104-134) | 0.73(0.6-0.86) | 161(140-189) | 0.88(0.74-1.05) | 1.41 (1 to 1.77) | <0.001 |
|  | 35-39 | 211(188-238) | 1.43(1.21-1.68) | 339(287-401) | 1.8(1.49-2.15) | 1.71 (1.45 to 1.94) | <0.001 |
|  | 40-44 | 274(249-302) | 2.22(1.86-2.59) | 375(321-438) | 2.71(2.24-3.28) | 1.14 (0.88 to 1.36) | <0.001 |
| **South Asia** | 15-19 | 356(308-414) | 0.09(0.08-0.11) | 816(560-1079) | 0.14(0.12-0.16) | 1.02 (0.92 to 1.1) | <0.001 |
|  | 20-24 | 576(506-652) | 0.23(0.2-0.26) | 1488(1044-1972) | 0.43(0.36-0.5) | 1.32 (1.27 to 1.37) | <0.001 |
|  | 25-29 | 905(781-1047) | 0.51(0.45-0.58) | 2419(1798-3020) | 0.91(0.77-1.06) | 1.2 (1.12 to 1.26) | <0.001 |
|  | 30-34 | 1449(1250-1657) | 0.93(0.82-1.06) | 4256(3258-5143) | 1.53(1.32-1.79) | 1.22 (1.16 to 1.27) | <0.001 |
|  | 35-39 | 2489(2141-2837) | 1.65(1.47-1.86) | 7146(5709-8491) | 2.89(2.45-3.41) | 1.03 (0.91 to 1.2) | <0.001 |
|  | 40-44 | 3541(3148-3946) | 1.99(1.82-2.2) | 10604(8762-12081) | 2.87(2.45-3.35) | 0.97 (0.91 to 1.03) | <0.001 |
| **Southeast Asia** | 15-19 | 57(46-70) | 0.33(0.28-0.38) | 71(60-83) | 0.46(0.32-0.61) | 0.18 (0.13 to 0.26) | <0.001 |
|  | 20-24 | 95(76-117) | 0.58(0.51-0.66) | 130(107-154) | 0.87(0.61-1.15) | 0.21 (0.16 to 0.28) | <0.001 |
|  | 25-29 | 158(129-191) | 1.05(0.91-1.22) | 259(214-308) | 1.51(1.12-1.89) | 0.43 (0.37 to 0.49) | <0.001 |
|  | 30-34 | 254(210-300) | 1.98(1.7-2.26) | 485(411-579) | 2.87(2.2-3.47) | 0.56 (0.5 to 0.61) | <0.001 |
|  | 35-39 | 404(342-477) | 3.86(3.32-4.4) | 942(781-1125) | 5.29(4.23-6.29) | 0.73 (0.68 to 0.76) | <0.001 |
|  | 40-44 | 481(401-560) | 6.65(5.91-7.42) | 1329(1101-1609) | 9.13(7.55-10.41) | 0.63 (0.59 to 0.66) | <0.001 |

AAPC: average annual percent change; ASR: age-standardized rate.

Supplementary Table 7**.** Number of cases and ASR of lip and oral cavity cancer deaths among young people (1990, 2021), with AAPC from 1990 to 2021, by age group.

| **Location** | **Age group** | **1990** | | **2021** | | **AAPC (95%CI)** | ***P*** |
| --- | --- | --- | --- | --- | --- | --- | --- |
|  |  | **Number (95%UI)** | **ASR (95%UI)** | **Number (95%UI)** | **ASR (95%UI)** |  |  |
| **Global** | 15-19 | 359(327-401) | 0.07(0.06-0.08) | 506(385-622) | 0.08(0.06-0.1) | 0.54 (0.45 to 0.6) | <0.001 |
|  | 20-24 | 570(527-622) | 0.12(0.11-0.13) | 915(705-1138) | 0.15(0.12-0.19) | 0.93 (0.85 to 0.98) | <0.001 |
|  | 25-29 | 840(762-920) | 0.19(0.17-0.21) | 1418(1116-1690) | 0.24(0.19-0.29) | 0.8 (0.72 to 0.86) | <0.001 |
|  | 30-34 | 1421(1297-1551) | 0.37(0.34-0.4) | 2601(2134-2999) | 0.43(0.35-0.5) | 0.46 (0.41 to 0.5) | <0.001 |
|  | 35-39 | 2527(2304-2727) | 0.72(0.65-0.77) | 4375(3697-4970) | 0.78(0.66-0.89) | 0.27 (0.23 to 0.29) | <0.001 |
|  | 40-44 | 4445(4149-4749) | 1.55(1.45-1.66) | 8064(7022-8907) | 1.61(1.4-1.78) | 0.14 (0.08 to 0.19) | 0.006 |
| **East Asia** | 15-19 | 55(48-64) | 0.04(0.04-0.05) | 19(15-24) | 0.03(0.02-0.03) | -1.68 (-1.75 to -1.61) | <0.001 |
|  | 20-24 | 74(64-87) | 0.05(0.05-0.06) | 35(29-43) | 0.05(0.04-0.06) | -0.55 (-0.68 to -0.45) | <0.001 |
|  | 25-29 | 87(74-101) | 0.08(0.06-0.09) | 64(52-76) | 0.07(0.06-0.08) | -0.23 (-0.35 to -0.11) | 0.002 |
|  | 30-34 | 176(152-202) | 0.19(0.17-0.22) | 215(179-261) | 0.17(0.14-0.21) | -0.42 (-0.56 to -0.3) | <0.001 |
|  | 35-39 | 319(271-366) | 0.34(0.29-0.39) | 334(279-398) | 0.3(0.25-0.36) | -0.35 (-0.44 to -0.27) | <0.001 |
|  | 40-44 | 447(382-519) | 0.64(0.55-0.75) | 593(495-719) | 0.62(0.52-0.75) | -0.06 (-0.18 to 0.04) | 0.207 |
| **High-income Asia Pacific** | 15-19 | 3(3-3) | 0.48(0.45-0.5) | 1(1-1) | 0.02(0.01-0.02) | -0.62 (-0.83 to -0.38) | <0.001 |
|  | 20-24 | 7(6-7) | 0.02(0.02-0.02) | 5(4-5) | 0.05(0.05-0.05) | 0.24 (0.03 to 0.47) | 0.030 |
|  | 25-29 | 13(12-14) | 0.05(0.04-0.05) | 10(9-11) | 0.1(0.09-0.11) | 0.13 (-0.16 to 0.49) | 0.356 |
|  | 30-34 | 22(20-24) | 0.1(0.09-0.11) | 17(16-19) | 0.17(0.15-0.18) | -0.27 (-0.75 to 0.19) | 0.195 |
|  | 35-39 | 36(33-39) | 0.17(0.16-0.19) | 35(32-39) | 0.3(0.28-0.33) | 0.37 (0.13 to 0.63) | 0.004 |
|  | 40-44 | 65(62-69) | 0.28(0.26-0.3) | 56(52-61) | 0.43(0.4-0.47) | -0.31 (-0.57 to -0.13) | 0.002 |
| **South Asia** | 15-19 | 198(173-228) | 0.18(0.16-0.21) | 360(254-469) | 0.2(0.14-0.27) | 0.27 (0.16 to 0.42) | 0.000 |
|  | 20-24 | 334(293-379) | 0.34(0.3-0.38) | 681(478-895) | 0.4(0.28-0.52) | 0.59 (0.54 to 0.63) | <0.001 |
|  | 25-29 | 487(419-563) | 0.56(0.49-0.65) | 1021(756-1269) | 0.64(0.47-0.79) | 0.42 (0.36 to 0.48) | <0.001 |
|  | 30-34 | 776(665-891) | 1.06(0.91-1.22) | 1767(1362-2138) | 1.19(0.92-1.44) | 0.41 (0.34 to 0.46) | <0.001 |
|  | 35-39 | 1280(1095-1460) | 1.98(1.7-2.26) | 2846(2258-3389) | 2.11(1.67-2.51) | 0.25 (0.16 to 0.33) | <0.001 |
|  | 40-44 | 2163(1925-2421) | 4.07(3.62-4.55) | 5248(4321-6045) | 4.52(3.72-5.21) | 0.33 (0.27 to 0.39) | <0.001 |
| **Southeast Asia** | 15-19 | 25(20-32) | 0.05(0.04-0.06) | 25(21-29) | 0.04(0.04-0.05) | -0.56 (-0.61 to -0.48) | <0.001 |
|  | 20-24 | 44(35-55) | 0.1(0.08-0.12) | 47(40-56) | 0.08(0.07-0.1) | -0.55 (-0.6 to -0.48) | <0.001 |
|  | 25-29 | 67(55-81) | 0.17(0.14-0.2) | 85(71-100) | 0.15(0.12-0.18) | -0.41 (-0.46 to -0.36) | <0.001 |
|  | 30-34 | 107(89-127) | 0.31(0.25-0.36) | 154(129-183) | 0.28(0.23-0.33) | -0.33 (-0.38 to -0.28) | <0.001 |
|  | 35-39 | 164(139-190) | 0.58(0.49-0.67) | 284(235-335) | 0.54(0.45-0.64) | -0.21 (-0.25 to -0.18) | <0.001 |
|  | 40-44 | 239(200-278) | 1.11(0.93-1.29) | 511(422-615) | 1.04(0.86-1.25) | -0.2 (-0.23 to -0.17) | <0.001 |

AAPC: average annual percent change; ASR: age-standardized rate.

Supplementary Table 8**.** Number of cases and ASR of lip and oral cavity cancer DALYs among young people (1990, 2021), with AAPC from 1990 to 2021, by age group.

| **Location** | **Age group** | **1990** | | **2021** | | **AAPC (95%CI)** | ***P*** |
| --- | --- | --- | --- | --- | --- | --- | --- |
|  |  | **Number (95%UI)** | **ASR (95%UI)** | **Number (95%UI)** | **ASR (95%UI)** |  |  |
| **Global** | 15-19 | 26320(23980-29414) | 5.07(4.62-5.66) | 37228(28340-45837) | 5.97(4.54-7.35) | 0.55 (0.46 to 0.61) | <0.001 |
|  | 20-24 | 39034(36099-42598) | 7.93(7.34-8.66) | 62868(48348-78272) | 10.53(8.1-13.11) | 0.93 (0.86 to 0.99) | <0.001 |
|  | 25-29 | 53550(48722-58563) | 12.1(11.01-13.23) | 90635(71302-107606) | 15.41(12.12-18.29) | 0.81 (0.72 to 0.87) | <0.001 |
|  | 30-34 | 83558(76280-90942) | 21.68(19.79-23.6) | 153615(125706-177235) | 25.41(20.8-29.32) | 0.47 (0.42 to 0.52) | <0.001 |
|  | 35-39 | 136224(124224-146951) | 38.67(35.27-41.72) | 237048(200416-268586) | 42.26(35.73-47.89) | 0.28 (0.25 to 0.31) | <0.001 |
|  | 40-44 | 216672(202548-231478) | 75.63(70.7-80.8) | 394235(342680-434234) | 78.81(68.5-86.8) | 0.15 (0.09 to 0.2) | 0.006 |
| **East Asia** | 15-19 | 14516(12663-16728) | 3.12(2.72-3.58) | 1452(1150-1762) | 1.87(1.48-2.27) | -1.62 (-1.7 to -1.56) | <0.001 |
|  | 20-24 | 22793(20039-25903) | 3.75(3.23-4.39) | 2463(2034-2965) | 3.22(2.66-3.87) | -0.5 (-0.64 to -0.4) | <0.001 |
|  | 25-29 | 30894(26572-35677) | 4.87(4.12-5.67) | 4121(3396-4909) | 4.57(3.76-5.44) | -0.18 (-0.3 to -0.06) | 0.007 |
|  | 30-34 | 45464(38974-52162) | 11.26(9.71-12.87) | 12885(10737-15592) | 10.32(8.6-12.49) | -0.35 (-0.49 to -0.24) | <0.001 |
|  | 35-39 | 68495(58627-78033) | 18.23(15.42-20.84) | 18438(15402-21897) | 16.8(14.03-19.95) | -0.29 (-0.37 to -0.21) | <0.001 |
|  | 40-44 | 104757(93187-117136) | 31.36(26.72-36.42) | 29366(24436-35566) | 30.78(25.62-37.28) | -0.02 (-0.13 to 0.08) | 0.753 |
| **High-income Asia Pacific** | 15-19 | 4066(3543-4671) | 1.41(1.27-1.58) | 101(94-109) | 1.21(1.12-1.3) | -0.55 (-0.76 to -0.32) | <0.001 |
|  | 20-24 | 5087(4387-5963) | 3.3(3.02-3.67) | 343(318-372) | 3.59(3.32-3.89) | 0.32 (0.11 to 0.53) | 0.006 |
|  | 25-29 | 5535(4688-6450) | 6.39(5.86-7.1) | 682(621-760) | 6.62(6.03-7.38) | 0.23 (-0.06 to 0.58) | 0.112 |
|  | 30-34 | 10313(8895-11791) | 10.53(9.6-11.58) | 1086(1000-1193) | 10.29(9.47-11.3) | -0.16 (-0.63 to 0.28) | 0.417 |
|  | 35-39 | 17182(14539-19646) | 15.42(14.29-16.7) | 2029(1850-2255) | 17.27(15.74-19.2) | 0.51 (0.27 to 0.77) | <0.001 |
|  | 40-44 | 21767(18549-25280) | 23.65(22.51-24.86) | 2869(2628-3114) | 21.95(20.1-23.81) | -0.24 (-0.48 to -0.06) | 0.014 |
| **South Asia** | 15-19 | 214(192-239) | 13.31(11.61-15.34) | 26440(18619-34450) | 14.98(10.55-19.52) | 0.37 (0.27 to 0.46) | <0.001 |
|  | 20-24 | 459(420-511) | 23.12(20.32-26.27) | 46646(32724-61386) | 27.23(19.1-35.84) | 0.6 (0.55 to 0.64) | <0.001 |
|  | 25-29 | 835(765-927) | 35.87(30.86-41.43) | 65028(48175-81018) | 40.63(30.1-50.62) | 0.43 (0.37 to 0.49) | <0.001 |
|  | 30-34 | 1328(1212-1462) | 62(53.15-71.13) | 103874(79796-125607) | 70.14(53.89-84.82) | 0.42 (0.36 to 0.48) | <0.001 |
|  | 35-39 | 1972(1827-2135) | 106.19(90.89-120.98) | 153173(121161-183606) | 113.43(89.73-135.97) | 0.27 (0.17 to 0.35) | <0.001 |
|  | 40-44 | 3248(3091-3413) | 196.84(175.1-220.1) | 255308(210706-294195) | 219.94(181.51-253.44) | 0.34 (0.28 to 0.4) | <0.001 |
| **Southeast Asia** | 15-19 | 1869(1507-2319) | 3.8(3.06-4.72) | 1824(1535-2164) | 3.22(2.71-3.82) | -0.55 (-0.6 to -0.47) | <0.001 |
|  | 20-24 | 3024(2427-3737) | 6.79(5.45-8.38) | 3267(2732-3862) | 5.8(4.85-6.86) | -0.54 (-0.59 to -0.47) | <0.001 |
|  | 25-29 | 4301(3517-5192) | 10.71(8.76-12.93) | 5417(4546-6412) | 9.53(8-11.28) | -0.4 (-0.45 to -0.35) | <0.001 |
|  | 30-34 | 6288(5230-7442) | 18.07(15.03-21.39) | 9123(7625-10802) | 16.52(13.8-19.55) | -0.31 (-0.37 to -0.26) | <0.001 |
|  | 35-39 | 8820(7475-10232) | 31.12(26.38-36.1) | 15378(12789-18220) | 29.39(24.45-34.83) | -0.19 (-0.23 to -0.16) | <0.001 |
|  | 40-44 | 11649(9738-13546) | 53.91(45.07-62.69) | 25024(20620-30098) | 51(42.02-61.34) | -0.18 (-0.22 to -0.15) | <0.001 |

AAPC: average annual percent change; ASR: age-standardized rate; DALYs: disability-adjusted life-years.

Supplementary Table 9**.** The projected case number and ASR of lip and oral cavity cancer among young people up to 2030

| **Year** | **Incidence** | | **Deaths** | | **DALYs** | |
| --- | --- | --- | --- | --- | --- | --- |
|  | **Number** | **ASR** | **Number** | **ASR** | **Number** | **ASR** |
| **East Asia** | | | | | | |
| 1992 | 2812 | 0.46688 | 1220 | 0.203356 | 66824 | 11.050157 |
| 1993 | 2932 | 0.473096 | 1250 | 0.202033 | 68229 | 10.969313 |
| 1994 | 3019 | 0.480632 | 1265 | 0.201256 | 68937 | 10.928587 |
| 1995 | 3113 | 0.484412 | 1284 | 0.198972 | 69729 | 10.797051 |
| 1996 | 3204 | 0.492321 | 1296 | 0.198087 | 70305 | 10.749462 |
| 1997 | 3295 | 0.500239 | 1301 | 0.196586 | 70500 | 10.662821 |
| 1998 | 3398 | 0.510417 | 1315 | 0.197342 | 71167 | 10.681516 |
| 1999 | 3466 | 0.51739 | 1320 | 0.197892 | 71413 | 10.690728 |
| 2000 | 3550 | 0.527202 | 1325 | 0.198487 | 71832 | 10.726748 |
| 2001 | 3575 | 0.524722 | 1306 | 0.193969 | 70896 | 10.488083 |
| 2002 | 3682 | 0.526933 | 1322 | 0.191361 | 71767 | 10.366132 |
| 2003 | 3928 | 0.543029 | 1385 | 0.193183 | 75241 | 10.503764 |
| 2004 | 4142 | 0.552373 | 1422 | 0.190522 | 76971 | 10.361749 |
| 2005 | 4378 | 0.564717 | 1453 | 0.187399 | 78293 | 10.182543 |
| 2006 | 4650 | 0.583719 | 1493 | 0.186517 | 80056 | 10.115449 |
| 2007 | 4868 | 0.601478 | 1522 | 0.186362 | 81373 | 10.102655 |
| 2008 | 4980 | 0.615286 | 1521 | 0.185716 | 81367 | 10.088256 |
| 2009 | 5038 | 0.629469 | 1507 | 0.185613 | 80777 | 10.118964 |
| 2010 | 5155 | 0.652015 | 1508 | 0.187859 | 80922 | 10.25363 |
| 2011 | 5286 | 0.678725 | 1507 | 0.19027 | 80883 | 10.391061 |
| 2012 | 5230 | 0.683448 | 1460 | 0.187283 | 78381 | 10.231905 |
| 2013 | 5248 | 0.697689 | 1435 | 0.18713 | 77125 | 10.228494 |
| 2014 | 5460 | 0.738206 | 1462 | 0.193674 | 78558 | 10.586171 |
| 2015 | 5389 | 0.742178 | 1430 | 0.193131 | 76919 | 10.560935 |
| 2016 | 5364 | 0.75377 | 1403 | 0.193902 | 75647 | 10.608748 |
| 2017 | 5406 | 0.776147 | 1370 | 0.194126 | 74136 | 10.635005 |
| 2018 | 5470 | 0.800002 | 1350 | 0.1956 | 73241 | 10.725449 |
| 2019 | 5468 | 0.80948 | 1318 | 0.193901 | 71635 | 10.644776 |
| 2020 | 5364 | 0.798379 | 1271 | 0.18869 | 69229 | 10.373889 |
| 2021 | 5409 | 0.805859 | 1261 | 0.187981 | 68723 | 10.339972 |
| 2022 | 6049 | 0.832617 | 1381 | 0.19043 | 75296 | 10.499634 |
| 2023 | 6180 | 0.845234 | 1391 | 0.190183 | 75710 | 10.502252 |
| 2024 | 6348 | 0.857801 | 1411 | 0.189921 | 76585 | 10.504603 |
| 2025 | 6489 | 0.86573 | 1430 | 0.189235 | 77551 | 10.505005 |
| 2026 | 6609 | 0.873791 | 1445 | 0.188583 | 78272 | 10.507361 |
| 2027 | 6676 | 0.88203 | 1447 | 0.187973 | 78405 | 10.511967 |
| 2028 | 6682 | 0.890387 | 1436 | 0.187384 | 77889 | 10.517878 |
| 2029 | 6646 | 0.89886 | 1416 | 0.186814 | 76941 | 10.525256 |
| 2030 | 6576 | 0.907549 | 1390 | 0.186295 | 75633 | 10.535484 |
| **High-income Asia Pacific** | | | | | | |
| 1992 | 750 | 0.872042 | 151 | 0.172462 | 8338 | 9.659134 |
| 1993 | 750 | 0.881529 | 150 | 0.173489 | 8269 | 9.686269 |
| 1994 | 779 | 0.927367 | 154 | 0.180817 | 8495 | 10.081618 |
| 1995 | 913 | 1.095506 | 173 | 0.205371 | 9605 | 11.488425 |
| 1996 | 985 | 1.184979 | 180 | 0.214605 | 10013 | 12.017148 |
| 1997 | 975 | 1.175212 | 174 | 0.208376 | 9713 | 11.693588 |
| 1998 | 1009 | 1.210019 | 177 | 0.211437 | 9912 | 11.878184 |
| 1999 | 1042 | 1.242274 | 181 | 0.214661 | 10130 | 12.070876 |
| 2000 | 1071 | 1.273061 | 183 | 0.216744 | 10269 | 12.201467 |
| 2001 | 1031 | 1.223022 | 175 | 0.206717 | 9842 | 11.669426 |
| 2002 | 1050 | 1.236363 | 175 | 0.205157 | 9838 | 11.587615 |
| 2003 | 1100 | 1.290435 | 179 | 0.208098 | 10025 | 11.765554 |
| 2004 | 1106 | 1.293823 | 173 | 0.201027 | 9721 | 11.39333 |
| 2005 | 1095 | 1.27771 | 164 | 0.190466 | 9256 | 10.83662 |
| 2006 | 1172 | 1.364511 | 167 | 0.193936 | 9451 | 11.071659 |
| 2007 | 1232 | 1.428753 | 171 | 0.19706 | 9664 | 11.279888 |
| 2008 | 1312 | 1.51387 | 177 | 0.203233 | 10039 | 11.667918 |
| 2009 | 1293 | 1.487162 | 173 | 0.197092 | 9777 | 11.320066 |
| 2010 | 1302 | 1.498082 | 172 | 0.195473 | 9706 | 11.240416 |
| 2011 | 1314 | 1.494565 | 172 | 0.193099 | 9685 | 11.060288 |
| 2012 | 1298 | 1.480973 | 169 | 0.189201 | 9482 | 10.836055 |
| 2013 | 1256 | 1.442563 | 162 | 0.181885 | 9077 | 10.425151 |
| 2014 | 1276 | 1.488747 | 162 | 0.184864 | 9130 | 10.640905 |
| 2015 | 1283 | 1.509389 | 162 | 0.185384 | 9072 | 10.657518 |
| 2016 | 1309 | 1.564783 | 164 | 0.191095 | 9199 | 10.981281 |
| 2017 | 1194 | 1.454991 | 149 | 0.177718 | 8388 | 10.215262 |
| 2018 | 1113 | 1.386709 | 139 | 0.169143 | 7806 | 9.724274 |
| 2019 | 1073 | 1.369038 | 133 | 0.166 | 7493 | 9.559463 |
| 2020 | 1064 | 1.390973 | 131 | 0.167865 | 7429 | 9.701837 |
| 2021 | 1023 | 1.363928 | 126 | 0.16355 | 7111 | 9.468342 |
| 2022 | 987 | 1.328471 | 121 | 0.159403 | 6854 | 9.213212 |
| 2023 | 956 | 1.306703 | 117 | 0.156226 | 6612 | 9.038333 |
| 2024 | 925 | 1.284942 | 113 | 0.153053 | 6378 | 8.863551 |
| 2025 | 900 | 1.270266 | 109 | 0.150797 | 6182 | 8.74029 |
| 2026 | 875 | 1.255576 | 106 | 0.148537 | 5997 | 8.616836 |
| 2027 | 853 | 1.24086 | 102 | 0.146271 | 5823 | 8.493023 |
| 2028 | 832 | 1.226114 | 99 | 0.143995 | 5661 | 8.368797 |
| 2029 | 813 | 1.211347 | 97 | 0.141713 | 5509 | 8.244269 |
| 2030 | 795 | 1.196564 | 94 | 0.139427 | 5364 | 8.119521 |
| **South Asia** | | | | | | |
| 1992 | 10042 | 2.255613 | 5614 | 1.264879 | 307073 | 68.229256 |
| 1993 | 10313 | 2.255558 | 5757 | 1.262986 | 315058 | 68.173864 |
| 1994 | 10501 | 2.234127 | 5862 | 1.250997 | 321088 | 67.593517 |
| 1995 | 10875 | 2.247848 | 6091 | 1.262587 | 333121 | 68.154109 |
| 1996 | 11195 | 2.262946 | 6276 | 1.272713 | 343766 | 68.779036 |
| 1997 | 11617 | 2.291817 | 6507 | 1.288224 | 356941 | 69.699978 |
| 1998 | 12134 | 2.33314 | 6779 | 1.308183 | 372169 | 70.842855 |
| 1999 | 12739 | 2.386187 | 7086 | 1.33228 | 389130 | 72.171663 |
| 2000 | 13194 | 2.405632 | 7297 | 1.33533 | 401007 | 72.409226 |
| 2001 | 13626 | 2.420327 | 7485 | 1.334104 | 411280 | 72.361629 |
| 2002 | 14072 | 2.436945 | 7688 | 1.335612 | 421992 | 72.409139 |
| 2003 | 14488 | 2.446631 | 7875 | 1.333978 | 432024 | 72.305448 |
| 2004 | 14862 | 2.447733 | 8033 | 1.326961 | 440893 | 71.974674 |
| 2005 | 15441 | 2.483323 | 8287 | 1.33656 | 454916 | 72.533471 |
| 2006 | 15872 | 2.49711 | 8453 | 1.333737 | 463479 | 72.313227 |
| 2007 | 16434 | 2.52967 | 8672 | 1.338583 | 474998 | 72.531696 |
| 2008 | 16994 | 2.557915 | 8866 | 1.337891 | 485841 | 72.55007 |
| 2009 | 17573 | 2.586501 | 9057 | 1.336111 | 496415 | 72.504065 |
| 2010 | 18165 | 2.614887 | 9247 | 1.333952 | 506758 | 72.398015 |
| 2011 | 18691 | 2.632573 | 9421 | 1.329749 | 516002 | 72.145234 |
| 2012 | 19314 | 2.664243 | 9630 | 1.331046 | 527207 | 72.206113 |
| 2013 | 20194 | 2.730195 | 9948 | 1.347506 | 544586 | 73.111029 |
| 2014 | 20674 | 2.740287 | 10057 | 1.335653 | 550622 | 72.486143 |
| 2015 | 21350 | 2.773425 | 10241 | 1.333195 | 560572 | 72.355212 |
| 2016 | 22395 | 2.851377 | 10602 | 1.352969 | 579864 | 73.394621 |
| 2017 | 23508 | 2.934057 | 11014 | 1.377982 | 602057 | 74.737916 |
| 2018 | 24009 | 2.936786 | 11135 | 1.365472 | 608487 | 74.064329 |
| 2019 | 24645 | 2.953403 | 11312 | 1.359041 | 617952 | 73.734452 |
| 2020 | 25947 | 3.047644 | 11738 | 1.382239 | 641021 | 75.01469 |
| 2021 | 26729 | 3.079166 | 11922 | 1.37679 | 650469 | 74.700083 |
| 2022 | 27521 | 3.091125 | 12236 | 1.376413 | 668554 | 74.866411 |
| 2023 | 28328 | 3.124918 | 12479 | 1.378194 | 681856 | 75.025832 |
| 2024 | 29143 | 3.158802 | 12724 | 1.38006 | 695172 | 75.189226 |
| 2025 | 29895 | 3.186311 | 12977 | 1.383412 | 708577 | 75.392311 |
| 2026 | 30636 | 3.213861 | 13222 | 1.38679 | 721542 | 75.5968 |
| 2027 | 31355 | 3.241434 | 13455 | 1.390173 | 733872 | 75.801646 |
| 2028 | 32045 | 3.268998 | 13669 | 1.393534 | 745331 | 76.005754 |
| 2029 | 32706 | 3.296568 | 13869 | 1.396893 | 756000 | 76.209935 |
| 2030 | 33347 | 3.324209 | 14057 | 1.400294 | 766077 | 76.416108 |
| **Southeast Asia** | | | | | | |
| 1992 | 1614 | 0.809189 | 705 | 0.356451 | 39103 | 19.406757 |
| 1993 | 1709 | 0.826396 | 740 | 0.360335 | 40959 | 19.629421 |
| 1994 | 1790 | 0.836552 | 770 | 0.362492 | 42610 | 19.752005 |
| 1995 | 1887 | 0.851967 | 808 | 0.367112 | 44642 | 20.006497 |
| 1996 | 1967 | 0.862215 | 838 | 0.369632 | 46273 | 20.155673 |
| 1997 | 2016 | 0.859272 | 858 | 0.36761 | 47290 | 20.044272 |
| 1998 | 2094 | 0.869206 | 887 | 0.369922 | 48816 | 20.160185 |
| 1999 | 2213 | 0.895248 | 930 | 0.377512 | 51130 | 20.591296 |
| 2000 | 2286 | 0.902981 | 954 | 0.37806 | 52430 | 20.630832 |
| 2001 | 2320 | 0.897162 | 964 | 0.373728 | 52878 | 20.376271 |
| 2002 | 2357 | 0.89371 | 974 | 0.36973 | 53293 | 20.143485 |
| 2003 | 2384 | 0.887685 | 981 | 0.365523 | 53622 | 19.905721 |
| 2004 | 2393 | 0.875963 | 981 | 0.359169 | 53550 | 19.548893 |
| 2005 | 2383 | 0.858461 | 974 | 0.350919 | 53118 | 19.08876 |
| 2006 | 2398 | 0.850775 | 970 | 0.344147 | 52887 | 18.724818 |
| 2007 | 2439 | 0.852792 | 974 | 0.340666 | 53097 | 18.535094 |
| 2008 | 2478 | 0.853283 | 979 | 0.336918 | 53319 | 18.33096 |
| 2009 | 2512 | 0.850764 | 982 | 0.332338 | 53470 | 18.080549 |
| 2010 | 2548 | 0.851228 | 984 | 0.328297 | 53545 | 17.86252 |
| 2011 | 2599 | 0.858918 | 994 | 0.328055 | 54063 | 17.851152 |
| 2012 | 2634 | 0.86068 | 998 | 0.32573 | 54262 | 17.718951 |
| 2013 | 2680 | 0.865369 | 1008 | 0.324891 | 54749 | 17.66764 |
| 2014 | 2743 | 0.875114 | 1021 | 0.325356 | 55450 | 17.690915 |
| 2015 | 2823 | 0.890403 | 1038 | 0.326995 | 56344 | 17.778327 |
| 2016 | 2899 | 0.904055 | 1053 | 0.327984 | 57139 | 17.832905 |
| 2017 | 2965 | 0.914163 | 1064 | 0.327885 | 57760 | 17.828415 |
| 2018 | 3039 | 0.926202 | 1078 | 0.3281 | 58485 | 17.848416 |
| 2019 | 3129 | 0.942498 | 1095 | 0.32951 | 59452 | 17.937063 |
| 2020 | 3142 | 0.93544 | 1090 | 0.323893 | 59096 | 17.625265 |
| 2021 | 3217 | 0.947772 | 1106 | 0.325083 | 60033 | 17.723221 |
| 2022 | 3243 | 0.964292 | 1086 | 0.321915 | 59377 | 17.688259 |
| 2023 | 3297 | 0.974467 | 1087 | 0.320216 | 59608 | 17.651914 |
| 2024 | 3347 | 0.984619 | 1087 | 0.318508 | 59764 | 17.615069 |
| 2025 | 3387 | 0.992291 | 1087 | 0.317403 | 59905 | 17.587971 |
| 2026 | 3425 | 0.999954 | 1088 | 0.316295 | 60022 | 17.560826 |
| 2027 | 3464 | 1.007617 | 1088 | 0.315191 | 60140 | 17.533863 |
| 2028 | 3502 | 1.015261 | 1088 | 0.31408 | 60261 | 17.506658 |
| 2029 | 3540 | 1.022879 | 1088 | 0.312961 | 60371 | 17.479028 |
| 2030 | 3577 | 1.0305 | 1088 | 0.311844 | 60458 | 17.451619 |

ASR: age-standardized rate; DALYs: age-standardized disability-adjusted life years.

Supplementary Table 10**.** Sensitivity analysis of the Nordpred APC model: Predictions of lip and oral cavity cancer among young people using original data, lower bound, and upper bound of case numbers.

| **Year** | **Case numbers of incidence** | | | **Case numbers of deaths** | | | **Case numbers of DALYs** | | |
| --- | --- | --- | --- | --- | --- | --- | --- | --- | --- |
|  | **Original Data Prediction** | **Lower Bound Data Prediction** | **Upper Bound Data Prediction** | **Original Data Prediction** | **Lower Bound Data Prediction** | **Upper Bound Data Prediction** | **Original Data Prediction** | **Lower Bound Data Prediction** | **Upper Bound Data Prediction** |
| **East Asia** | | | | | | | | | |
| 1992 | 2812 | 2364 | 3314 | 1220 | 1019 | 1443 | 66824 | 55840 | 79058 |
| 1993 | 2932 | 2472 | 3462 | 1250 | 1050 | 1486 | 68229 | 57312 | 81166 |
| 1994 | 3019 | 2591 | 3500 | 1265 | 1085 | 1475 | 68937 | 59078 | 80575 |
| 1995 | 3113 | 2680 | 3599 | 1284 | 1105 | 1486 | 69729 | 59919 | 80877 |
| 1996 | 3204 | 2764 | 3713 | 1296 | 1112 | 1500 | 70305 | 60204 | 81578 |
| 1997 | 3295 | 2843 | 3791 | 1301 | 1127 | 1488 | 70500 | 60974 | 80806 |
| 1998 | 3398 | 2962 | 3922 | 1315 | 1147 | 1512 | 71167 | 62020 | 81864 |
| 1999 | 3466 | 3019 | 3981 | 1320 | 1150 | 1517 | 71413 | 62229 | 82036 |
| 2000 | 3550 | 3064 | 4102 | 1325 | 1149 | 1517 | 71832 | 62319 | 82366 |
| 2001 | 3575 | 3097 | 4145 | 1306 | 1131 | 1498 | 70896 | 61383 | 81373 |
| 2002 | 3682 | 3235 | 4216 | 1322 | 1168 | 1507 | 71767 | 63325 | 81873 |
| 2003 | 3928 | 3437 | 4498 | 1385 | 1223 | 1573 | 75241 | 66392 | 85542 |
| 2004 | 4142 | 3649 | 4701 | 1422 | 1267 | 1607 | 76971 | 68446 | 87046 |
| 2005 | 4378 | 3835 | 4973 | 1453 | 1287 | 1641 | 78293 | 69303 | 88589 |
| 2006 | 4650 | 4067 | 5303 | 1493 | 1322 | 1690 | 80056 | 70854 | 90708 |
| 2007 | 4868 | 4220 | 5589 | 1522 | 1336 | 1742 | 81373 | 71373 | 93169 |
| 2008 | 4980 | 4324 | 5744 | 1521 | 1338 | 1742 | 81367 | 71524 | 93093 |
| 2009 | 5038 | 4435 | 5732 | 1507 | 1334 | 1706 | 80777 | 71545 | 91373 |
| 2010 | 5155 | 4511 | 5864 | 1508 | 1339 | 1708 | 80922 | 71783 | 91622 |
| 2011 | 5286 | 4600 | 6071 | 1507 | 1329 | 1712 | 80883 | 71259 | 91963 |
| 2012 | 5230 | 4492 | 6069 | 1460 | 1256 | 1685 | 78381 | 67457 | 90461 |
| 2013 | 5248 | 4483 | 6075 | 1435 | 1237 | 1651 | 77125 | 66312 | 88670 |
| 2014 | 5460 | 4561 | 6449 | 1462 | 1224 | 1723 | 78558 | 65805 | 92489 |
| 2015 | 5389 | 4401 | 6462 | 1430 | 1169 | 1708 | 76919 | 62858 | 92007 |
| 2016 | 5364 | 4354 | 6521 | 1403 | 1137 | 1709 | 75647 | 61444 | 92000 |
| 2017 | 5406 | 4373 | 6529 | 1370 | 1113 | 1648 | 74136 | 60096 | 89188 |
| 2018 | 5470 | 4389 | 6678 | 1350 | 1087 | 1641 | 73241 | 58802 | 89276 |
| 2019 | 5468 | 4307 | 6790 | 1318 | 1051 | 1626 | 71635 | 57128 | 88555 |
| 2020 | 5364 | 4257 | 6712 | 1271 | 1012 | 1580 | 69229 | 55055 | 86222 |
| 2021 | 5409 | 4261 | 6851 | 1261 | 1007 | 1585 | 68723 | 54659 | 86306 |
| 2022 | 6049 | 4684 | 7682 | 1381 | 1080 | 1730 | 75296 | 58860 | 94943 |
| 2023 | 6180 | 4746 | 7908 | 1391 | 1078 | 1752 | 75710 | 58724 | 96197 |
| 2024 | 6348 | 4837 | 8181 | 1411 | 1085 | 1787 | 76585 | 58949 | 98040 |
| 2025 | 6489 | 4911 | 8416 | 1430 | 1092 | 1819 | 77551 | 59285 | 99895 |
| 2026 | 6609 | 4966 | 8623 | 1445 | 1095 | 1846 | 78272 | 59422 | 101454 |
| 2027 | 6676 | 4982 | 8764 | 1447 | 1089 | 1857 | 78405 | 59102 | 102263 |
| 2028 | 6682 | 4952 | 8825 | 1436 | 1072 | 1852 | 77889 | 58292 | 102228 |
| 2029 | 6646 | 4892 | 8830 | 1416 | 1050 | 1836 | 76941 | 57169 | 101617 |
| 2030 | 6576 | 4807 | 8787 | 1390 | 1022 | 1810 | 75633 | 55790 | 100514 |
| **High-income Asia Pacific** | | | | | | | | | |
| 1992 | 750 | 644 | 877 | 151 | 139 | 165 | 8338 | 7663 | 9169 |
| 1993 | 750 | 643 | 873 | 150 | 137 | 164 | 8269 | 7574 | 9101 |
| 1994 | 779 | 667 | 906 | 154 | 142 | 168 | 8495 | 7803 | 9334 |
| 1995 | 913 | 783 | 1063 | 173 | 160 | 188 | 9605 | 8888 | 10471 |
| 1996 | 985 | 841 | 1146 | 180 | 167 | 195 | 10013 | 9293 | 10903 |
| 1997 | 975 | 833 | 1137 | 174 | 161 | 189 | 9713 | 8995 | 10580 |
| 1998 | 1009 | 865 | 1178 | 177 | 165 | 193 | 9912 | 9196 | 10795 |
| 1999 | 1042 | 893 | 1217 | 181 | 168 | 197 | 10130 | 9404 | 11005 |
| 2000 | 1071 | 918 | 1248 | 183 | 171 | 199 | 10269 | 9543 | 11137 |
| 2001 | 1031 | 882 | 1201 | 175 | 163 | 190 | 9842 | 9136 | 10671 |
| 2002 | 1050 | 893 | 1226 | 175 | 163 | 190 | 9838 | 9153 | 10674 |
| 2003 | 1100 | 937 | 1287 | 179 | 167 | 193 | 10025 | 9371 | 10830 |
| 2004 | 1106 | 937 | 1294 | 173 | 162 | 187 | 9721 | 9072 | 10522 |
| 2005 | 1095 | 927 | 1278 | 164 | 154 | 178 | 9256 | 8636 | 10006 |
| 2006 | 1172 | 986 | 1379 | 167 | 157 | 180 | 9451 | 8829 | 10203 |
| 2007 | 1232 | 1035 | 1451 | 171 | 160 | 183 | 9664 | 9041 | 10416 |
| 2008 | 1312 | 1099 | 1559 | 177 | 167 | 189 | 10039 | 9430 | 10784 |
| 2009 | 1293 | 1082 | 1543 | 173 | 162 | 185 | 9777 | 9148 | 10534 |
| 2010 | 1302 | 1086 | 1556 | 172 | 161 | 184 | 9706 | 9088 | 10437 |
| 2011 | 1314 | 1093 | 1576 | 172 | 162 | 184 | 9685 | 9064 | 10413 |
| 2012 | 1298 | 1077 | 1572 | 169 | 159 | 181 | 9482 | 8853 | 10175 |
| 2013 | 1256 | 1043 | 1522 | 162 | 152 | 173 | 9077 | 8451 | 9767 |
| 2014 | 1276 | 1055 | 1548 | 162 | 152 | 174 | 9130 | 8496 | 9832 |
| 2015 | 1283 | 1058 | 1556 | 162 | 151 | 173 | 9072 | 8417 | 9786 |
| 2016 | 1309 | 1074 | 1591 | 164 | 153 | 176 | 9199 | 8535 | 9926 |
| 2017 | 1194 | 979 | 1448 | 149 | 139 | 162 | 8388 | 7738 | 9118 |
| 2018 | 1113 | 910 | 1353 | 139 | 128 | 152 | 7806 | 7157 | 8562 |
| 2019 | 1073 | 872 | 1307 | 133 | 122 | 147 | 7493 | 6819 | 8294 |
| 2020 | 1064 | 863 | 1295 | 131 | 120 | 146 | 7429 | 6733 | 8255 |
| 2021 | 1023 | 824 | 1246 | 126 | 114 | 140 | 7111 | 6416 | 7969 |
| 2022 | 987 | 797 | 1201 | 121 | 110 | 137 | 6854 | 6178 | 7708 |
| 2023 | 956 | 770 | 1162 | 117 | 105 | 134 | 6612 | 5934 | 7485 |
| 2024 | 925 | 743 | 1125 | 113 | 101 | 130 | 6378 | 5699 | 7269 |
| 2025 | 900 | 721 | 1095 | 109 | 98 | 127 | 6182 | 5509 | 7077 |
| 2026 | 875 | 700 | 1066 | 106 | 94 | 124 | 5997 | 5330 | 6895 |
| 2027 | 853 | 680 | 1039 | 102 | 91 | 121 | 5823 | 5162 | 6726 |
| 2028 | 832 | 662 | 1015 | 99 | 88 | 118 | 5661 | 5004 | 6570 |
| 2029 | 813 | 646 | 992 | 97 | 85 | 116 | 5509 | 4855 | 6424 |
| 2030 | 795 | 630 | 970 | 94 | 83 | 114 | 5364 | 4713 | 6286 |
| **South Asia** | | | | | | | | | |
| 1992 | 10042 | 8353 | 11821 | 5614 | 4670 | 6600 | 307073 | 255194 | 361220 |
| 1993 | 10313 | 8566 | 12076 | 5757 | 4781 | 6753 | 315058 | 261421 | 370063 |
| 1994 | 10501 | 8838 | 12276 | 5862 | 4953 | 6849 | 321088 | 271180 | 375716 |
| 1995 | 10875 | 9197 | 12670 | 6091 | 5160 | 7075 | 333121 | 281973 | 387706 |
| 1996 | 11195 | 9486 | 13109 | 6276 | 5329 | 7337 | 343766 | 291495 | 402378 |
| 1997 | 11617 | 9920 | 13452 | 6507 | 5581 | 7515 | 356941 | 305526 | 412746 |
| 1998 | 12134 | 10430 | 14027 | 6779 | 5851 | 7793 | 372169 | 320574 | 428730 |
| 1999 | 12739 | 11040 | 14614 | 7086 | 6155 | 8110 | 389130 | 337506 | 445904 |
| 2000 | 13194 | 11425 | 15129 | 7297 | 6344 | 8357 | 401007 | 347917 | 459831 |
| 2001 | 13626 | 11810 | 15609 | 7485 | 6485 | 8548 | 411280 | 356014 | 470102 |
| 2002 | 14072 | 12263 | 16112 | 7688 | 6712 | 8769 | 421992 | 367940 | 482222 |
| 2003 | 14488 | 12584 | 16582 | 7875 | 6871 | 8962 | 432024 | 376563 | 492513 |
| 2004 | 14862 | 12953 | 16949 | 8033 | 7049 | 9144 | 440893 | 386111 | 502976 |
| 2005 | 15441 | 13407 | 17596 | 8287 | 7221 | 9436 | 454916 | 395507 | 518818 |
| 2006 | 15872 | 13872 | 18184 | 8453 | 7419 | 9643 | 463479 | 406227 | 529938 |
| 2007 | 16434 | 14378 | 18753 | 8672 | 7627 | 9825 | 474998 | 416727 | 540108 |
| 2008 | 16994 | 14885 | 19410 | 8866 | 7762 | 10096 | 485841 | 424595 | 554874 |
| 2009 | 17573 | 15200 | 20273 | 9057 | 7831 | 10406 | 496415 | 428768 | 571576 |
| 2010 | 18165 | 15630 | 20921 | 9247 | 7990 | 10605 | 506758 | 437168 | 583226 |
| 2011 | 18691 | 15989 | 21735 | 9421 | 8105 | 10915 | 516002 | 442397 | 599112 |
| 2012 | 19314 | 16424 | 22390 | 9630 | 8206 | 11161 | 527207 | 447973 | 612520 |
| 2013 | 20194 | 17048 | 23577 | 9948 | 8418 | 11604 | 544586 | 458603 | 638044 |
| 2014 | 20674 | 17025 | 24373 | 10057 | 8287 | 11825 | 550622 | 452347 | 649591 |
| 2015 | 21350 | 17080 | 25505 | 10241 | 8196 | 12271 | 560572 | 447266 | 674228 |
| 2016 | 22395 | 17900 | 26752 | 10602 | 8450 | 12664 | 579864 | 459934 | 695653 |
| 2017 | 23508 | 18804 | 27986 | 11014 | 8847 | 13129 | 602057 | 479965 | 721001 |
| 2018 | 24009 | 18819 | 28914 | 11135 | 8809 | 13373 | 608487 | 477597 | 734742 |
| 2019 | 24645 | 18904 | 30200 | 11312 | 8704 | 13856 | 617952 | 472151 | 761178 |
| 2020 | 25947 | 19710 | 32176 | 11738 | 8916 | 14524 | 641021 | 483822 | 797553 |
| 2021 | 26729 | 20231 | 33105 | 11922 | 9087 | 14762 | 650469 | 492290 | 809889 |
| 2022 | 27521 | 20457 | 34658 | 12236 | 9179 | 15167 | 668554 | 496085 | 840759 |
| 2023 | 28328 | 20792 | 36022 | 12479 | 9252 | 15553 | 681856 | 499401 | 864618 |
| 2024 | 29143 | 21123 | 37407 | 12724 | 9321 | 15942 | 695172 | 502505 | 888710 |
| 2025 | 29895 | 21463 | 38658 | 12977 | 9426 | 16322 | 708577 | 507057 | 911706 |
| 2026 | 30636 | 21787 | 39902 | 13222 | 9522 | 16693 | 721542 | 511115 | 934323 |
| 2027 | 31355 | 22091 | 41129 | 13455 | 9607 | 17051 | 733872 | 514545 | 956311 |
| 2028 | 32045 | 22366 | 42326 | 13669 | 9675 | 17390 | 745331 | 517188 | 977364 |
| 2029 | 32706 | 22615 | 43494 | 13869 | 9730 | 17710 | 756000 | 519122 | 997554 |
| 2030 | 33347 | 22846 | 44641 | 14057 | 9775 | 18018 | 766077 | 520522 | 1017098 |
| **Southeast Asia** | | | | | | | | | |
| 1992 | 1614 | 1308 | 1959 | 705 | 576 | 850 | 39103 | 31865 | 47244 |
| 1993 | 1709 | 1387 | 2079 | 740 | 606 | 893 | 40959 | 33473 | 49586 |
| 1994 | 1790 | 1453 | 2192 | 770 | 632 | 938 | 42610 | 34905 | 51916 |
| 1995 | 1887 | 1534 | 2316 | 808 | 666 | 982 | 44642 | 36647 | 54317 |
| 1996 | 1967 | 1598 | 2416 | 838 | 687 | 1016 | 46273 | 37783 | 56204 |
| 1997 | 2016 | 1646 | 2475 | 858 | 705 | 1037 | 47290 | 38787 | 57266 |
| 1998 | 2094 | 1702 | 2586 | 887 | 730 | 1074 | 48816 | 40050 | 59194 |
| 1999 | 2213 | 1792 | 2732 | 930 | 764 | 1120 | 51130 | 41894 | 61753 |
| 2000 | 2286 | 1839 | 2821 | 954 | 782 | 1158 | 52430 | 42812 | 63761 |
| 2001 | 2320 | 1868 | 2861 | 964 | 793 | 1173 | 52878 | 43364 | 64397 |
| 2002 | 2357 | 1896 | 2926 | 974 | 800 | 1181 | 53293 | 43620 | 64786 |
| 2003 | 2384 | 1934 | 2935 | 981 | 811 | 1180 | 53622 | 44196 | 64673 |
| 2004 | 2393 | 1955 | 2912 | 981 | 817 | 1172 | 53550 | 44468 | 64244 |
| 2005 | 2383 | 1966 | 2877 | 974 | 818 | 1159 | 53118 | 44466 | 63315 |
| 2006 | 2398 | 1982 | 2887 | 970 | 821 | 1153 | 52887 | 44680 | 62800 |
| 2007 | 2439 | 2034 | 2931 | 974 | 823 | 1157 | 53097 | 44723 | 63016 |
| 2008 | 2478 | 2066 | 2987 | 979 | 831 | 1159 | 53319 | 45182 | 63255 |
| 2009 | 2512 | 2089 | 3008 | 982 | 825 | 1166 | 53470 | 44859 | 63615 |
| 2010 | 2548 | 2132 | 3041 | 984 | 832 | 1160 | 53545 | 45240 | 63184 |
| 2011 | 2599 | 2168 | 3109 | 994 | 838 | 1170 | 54063 | 45529 | 63808 |
| 2012 | 2634 | 2197 | 3145 | 998 | 840 | 1184 | 54262 | 45579 | 64428 |
| 2013 | 2680 | 2209 | 3219 | 1008 | 842 | 1193 | 54749 | 45766 | 64780 |
| 2014 | 2743 | 2269 | 3295 | 1021 | 854 | 1217 | 55450 | 46301 | 66256 |
| 2015 | 2823 | 2337 | 3408 | 1038 | 867 | 1235 | 56344 | 46976 | 67181 |
| 2016 | 2899 | 2384 | 3492 | 1053 | 867 | 1260 | 57139 | 47081 | 68522 |
| 2017 | 2965 | 2451 | 3577 | 1064 | 886 | 1269 | 57760 | 47999 | 68916 |
| 2018 | 3039 | 2483 | 3698 | 1078 | 888 | 1291 | 58485 | 48170 | 70040 |
| 2019 | 3129 | 2534 | 3853 | 1095 | 895 | 1332 | 59452 | 48672 | 72299 |
| 2020 | 3142 | 2501 | 3888 | 1090 | 884 | 1337 | 59096 | 47936 | 72494 |
| 2021 | 3217 | 2541 | 4008 | 1106 | 887 | 1356 | 60033 | 48131 | 73559 |
| 2022 | 3243 | 2546 | 4027 | 1086 | 891 | 1340 | 59377 | 48343 | 73104 |
| 2023 | 3297 | 2565 | 4109 | 1087 | 893 | 1350 | 59608 | 48473 | 73774 |
| 2024 | 3347 | 2581 | 4186 | 1087 | 893 | 1357 | 59764 | 48540 | 74355 |
| 2025 | 3387 | 2594 | 4248 | 1087 | 895 | 1364 | 59905 | 48614 | 74792 |
| 2026 | 3425 | 2606 | 4308 | 1088 | 896 | 1370 | 60022 | 48667 | 75201 |
| 2027 | 3464 | 2617 | 4369 | 1088 | 897 | 1376 | 60140 | 48720 | 75613 |
| 2028 | 3502 | 2629 | 4430 | 1088 | 898 | 1383 | 60261 | 48777 | 76031 |
| 2029 | 3540 | 2640 | 4489 | 1088 | 899 | 1389 | 60371 | 48822 | 76436 |
| 2030 | 3577 | 2650 | 4548 | 1088 | 900 | 1395 | 60458 | 48850 | 76814 |

ASR: age-standardized rate; DALYs: age-standardized disability-adjusted life years.

Supplementary Table 11**.** Sensitivity analysis of the Nordpred APC model: Predictions of lip and oral cavity cancer among young people using original data, lower bound, and upper bound of ASR.

| **Year** | **ASIR** | | | **ASMR** | | | **Age-standardized DALYs rate** | | |
| --- | --- | --- | --- | --- | --- | --- | --- | --- | --- |
|  | **Original Data Prediction** | **Lower Bound Data Prediction** | **Upper Bound Data Prediction** | **Original Data Prediction** | **Lower Bound Data Prediction** | **Upper Bound Data Prediction** | **Original Data Prediction** | **Lower Bound Data Prediction** | **Upper Bound Data Prediction** |
| **East Asia** | | | | | | | | | |
| 1992 | 0.46688 | 0.392721 | 0.549831 | 0.203356 | 0.169904 | 0.240523 | 11.050157 | 9.237182 | 13.062929 |
| 1993 | 0.473096 | 0.399223 | 0.558191 | 0.202033 | 0.169743 | 0.239916 | 10.969313 | 9.221172 | 13.036533 |
| 1994 | 0.480632 | 0.412792 | 0.55668 | 0.201256 | 0.172788 | 0.234508 | 10.928587 | 9.37132 | 12.759801 |
| 1995 | 0.484412 | 0.41753 | 0.559418 | 0.198972 | 0.171467 | 0.230009 | 10.797051 | 9.290013 | 12.505731 |
| 1996 | 0.492321 | 0.425108 | 0.569582 | 0.198087 | 0.170106 | 0.228909 | 10.749462 | 9.215469 | 12.452707 |
| 1997 | 0.500239 | 0.43188 | 0.574839 | 0.196586 | 0.170475 | 0.224657 | 10.662821 | 9.229623 | 12.208258 |
| 1998 | 0.510417 | 0.445434 | 0.58819 | 0.197342 | 0.172367 | 0.226577 | 10.681516 | 9.320412 | 12.266703 |
| 1999 | 0.51739 | 0.45117 | 0.593764 | 0.197892 | 0.172703 | 0.227076 | 10.690728 | 9.328823 | 12.264054 |
| 2000 | 0.527202 | 0.455267 | 0.60862 | 0.198487 | 0.17228 | 0.226989 | 10.726748 | 9.313788 | 12.28272 |
| 2001 | 0.524722 | 0.454744 | 0.607144 | 0.193969 | 0.16792 | 0.222021 | 10.488083 | 9.083956 | 12.016923 |
| 2002 | 0.526933 | 0.462947 | 0.603403 | 0.191361 | 0.168955 | 0.218279 | 10.366132 | 9.141342 | 11.826448 |
| 2003 | 0.543029 | 0.474927 | 0.621781 | 0.193183 | 0.170519 | 0.219408 | 10.503764 | 9.265032 | 11.945986 |
| 2004 | 0.552373 | 0.486311 | 0.627099 | 0.190522 | 0.169583 | 0.215338 | 10.361749 | 9.205409 | 11.722332 |
| 2005 | 0.564717 | 0.494196 | 0.641866 | 0.187399 | 0.165787 | 0.211811 | 10.182543 | 9.003448 | 11.527642 |
| 2006 | 0.583719 | 0.509578 | 0.666553 | 0.186517 | 0.164831 | 0.211338 | 10.115449 | 8.93383 | 11.476244 |
| 2007 | 0.601478 | 0.520628 | 0.691234 | 0.186362 | 0.163321 | 0.213482 | 10.102655 | 8.848427 | 11.575853 |
| 2008 | 0.615286 | 0.533752 | 0.709778 | 0.185716 | 0.163229 | 0.212648 | 10.088256 | 8.86066 | 11.54577 |
| 2009 | 0.629469 | 0.553708 | 0.716761 | 0.185613 | 0.164355 | 0.210179 | 10.118964 | 8.961141 | 11.451447 |
| 2010 | 0.652015 | 0.570255 | 0.741994 | 0.187859 | 0.166725 | 0.212836 | 10.25363 | 9.090236 | 11.61221 |
| 2011 | 0.678725 | 0.590479 | 0.779961 | 0.19027 | 0.16774 | 0.216271 | 10.391061 | 9.149778 | 11.818615 |
| 2012 | 0.683448 | 0.586496 | 0.79392 | 0.187283 | 0.161119 | 0.216261 | 10.231905 | 8.800851 | 11.811841 |
| 2013 | 0.697689 | 0.595482 | 0.807435 | 0.18713 | 0.161225 | 0.215289 | 10.228494 | 8.793213 | 11.763921 |
| 2014 | 0.738206 | 0.616341 | 0.871264 | 0.193674 | 0.16233 | 0.228258 | 10.586171 | 8.873417 | 12.464612 |
| 2015 | 0.742178 | 0.605326 | 0.890054 | 0.193131 | 0.157902 | 0.230825 | 10.560935 | 8.625302 | 12.641503 |
| 2016 | 0.75377 | 0.611131 | 0.917515 | 0.193902 | 0.156961 | 0.236035 | 10.608748 | 8.608721 | 12.897496 |
| 2017 | 0.776147 | 0.627363 | 0.938197 | 0.194126 | 0.157627 | 0.233457 | 10.635005 | 8.618424 | 12.798858 |
| 2018 | 0.800002 | 0.641256 | 0.977302 | 0.1956 | 0.157347 | 0.237824 | 10.725449 | 8.603475 | 13.085429 |
| 2019 | 0.80948 | 0.637048 | 1.005855 | 0.193901 | 0.154496 | 0.239479 | 10.644776 | 8.47824 | 13.167395 |
| 2020 | 0.798379 | 0.633004 | 0.9996 | 0.18869 | 0.150136 | 0.234645 | 10.373889 | 8.238776 | 12.928547 |
| 2021 | 0.805859 | 0.634206 | 1.020625 | 0.187981 | 0.149964 | 0.236295 | 10.339972 | 8.210582 | 12.986638 |
| 2022 | 0.832617 | 0.64373 | 1.05863 | 0.19043 | 0.148553 | 0.238784 | 10.499634 | 8.191109 | 13.255091 |
| 2023 | 0.845234 | 0.647646 | 1.083361 | 0.190183 | 0.147012 | 0.239957 | 10.502252 | 8.125826 | 13.363205 |
| 2024 | 0.857801 | 0.651523 | 1.108024 | 0.189921 | 0.145459 | 0.241112 | 10.504603 | 8.060323 | 13.47094 |
| 2025 | 0.86573 | 0.65252 | 1.125801 | 0.189235 | 0.143804 | 0.241463 | 10.505005 | 8.002786 | 13.557972 |
| 2026 | 0.873791 | 0.653626 | 1.143743 | 0.188583 | 0.142177 | 0.241856 | 10.507361 | 7.946807 | 13.647465 |
| 2027 | 0.88203 | 0.654874 | 1.161907 | 0.187973 | 0.140583 | 0.242301 | 10.511967 | 7.892579 | 13.739839 |
| 2028 | 0.890387 | 0.656215 | 1.18022 | 0.187384 | 0.139005 | 0.24277 | 10.517878 | 7.83934 | 13.833891 |
| 2029 | 0.89886 | 0.657648 | 1.198675 | 0.186814 | 0.137443 | 0.243263 | 10.525256 | 7.787202 | 13.929815 |
| 2030 | 0.907549 | 0.659247 | 1.217402 | 0.186295 | 0.135919 | 0.243819 | 10.535484 | 7.737187 | 14.029441 |
| **High-income Asia Pacific** | | | | | | | | | |
| 1992 | 0.872042 | 0.748454 | 1.021442 | 0.172462 | 0.158547 | 0.189113 | 9.659134 | 8.854729 | 10.654661 |
| 1993 | 0.881529 | 0.754058 | 1.027477 | 0.173489 | 0.159071 | 0.190398 | 9.686269 | 8.854634 | 10.689095 |
| 1994 | 0.927367 | 0.792912 | 1.078802 | 0.180817 | 0.166398 | 0.198344 | 10.081618 | 9.245461 | 11.098026 |
| 1995 | 1.095506 | 0.938176 | 1.275482 | 0.205371 | 0.190385 | 0.223536 | 11.488425 | 10.621013 | 12.538056 |
| 1996 | 1.184979 | 1.011947 | 1.379817 | 0.214605 | 0.19887 | 0.233005 | 12.017148 | 11.145097 | 13.096522 |
| 1997 | 1.175212 | 1.003838 | 1.371206 | 0.208376 | 0.19306 | 0.226787 | 11.693588 | 10.823611 | 12.743682 |
| 1998 | 1.210019 | 1.036518 | 1.41356 | 0.211437 | 0.196105 | 0.230028 | 11.878184 | 11.016391 | 12.940478 |
| 1999 | 1.242274 | 1.064482 | 1.451003 | 0.214661 | 0.199361 | 0.232931 | 12.070876 | 11.202338 | 13.11656 |
| 2000 | 1.273061 | 1.090055 | 1.483005 | 0.216744 | 0.201608 | 0.234836 | 12.201467 | 11.336893 | 13.234783 |
| 2001 | 1.223022 | 1.045648 | 1.424544 | 0.206717 | 0.192239 | 0.223654 | 11.669426 | 10.833369 | 12.652432 |
| 2002 | 1.236363 | 1.050831 | 1.444047 | 0.205157 | 0.191092 | 0.222146 | 11.587615 | 10.781058 | 12.571164 |
| 2003 | 1.290435 | 1.097668 | 1.510666 | 0.208098 | 0.19443 | 0.224586 | 11.765554 | 10.997744 | 12.710296 |
| 2004 | 1.293823 | 1.095304 | 1.514488 | 0.201027 | 0.187873 | 0.217356 | 11.39333 | 10.632868 | 12.330969 |
| 2005 | 1.27771 | 1.080858 | 1.492793 | 0.190466 | 0.177934 | 0.205776 | 10.83662 | 10.111016 | 11.714758 |
| 2006 | 1.364511 | 1.1475 | 1.605737 | 0.193936 | 0.181479 | 0.208795 | 11.071659 | 10.342955 | 11.952635 |
| 2007 | 1.428753 | 1.198106 | 1.683878 | 0.19706 | 0.18459 | 0.211634 | 11.279888 | 10.550886 | 12.161511 |
| 2008 | 1.51387 | 1.266487 | 1.799778 | 0.203233 | 0.191187 | 0.217488 | 11.667918 | 10.957406 | 12.540122 |
| 2009 | 1.487162 | 1.242617 | 1.775642 | 0.197092 | 0.185146 | 0.211491 | 11.320066 | 10.58759 | 12.207134 |
| 2010 | 1.498082 | 1.248462 | 1.792717 | 0.195473 | 0.183711 | 0.209446 | 11.240416 | 10.521322 | 12.098656 |
| 2011 | 1.494565 | 1.24065 | 1.795104 | 0.193099 | 0.181307 | 0.207003 | 11.060288 | 10.342729 | 11.911738 |
| 2012 | 1.480973 | 1.226582 | 1.795982 | 0.189201 | 0.177517 | 0.202556 | 10.836055 | 10.108898 | 11.64755 |
| 2013 | 1.442563 | 1.196112 | 1.750224 | 0.181885 | 0.170171 | 0.195275 | 10.425151 | 9.697921 | 11.239764 |
| 2014 | 1.488747 | 1.228475 | 1.80825 | 0.184864 | 0.172855 | 0.198352 | 10.640905 | 9.892509 | 11.481426 |
| 2015 | 1.509389 | 1.243203 | 1.833459 | 0.185384 | 0.172897 | 0.199272 | 10.657518 | 9.878718 | 11.521984 |
| 2016 | 1.564783 | 1.282484 | 1.904358 | 0.191095 | 0.178189 | 0.205453 | 10.981281 | 10.178722 | 11.874105 |
| 2017 | 1.454991 | 1.19174 | 1.76495 | 0.177718 | 0.164647 | 0.192929 | 10.215262 | 9.416123 | 11.130382 |
| 2018 | 1.386709 | 1.132814 | 1.685938 | 0.169143 | 0.155734 | 0.185344 | 9.724274 | 8.90982 | 10.691168 |
| 2019 | 1.369038 | 1.112306 | 1.668131 | 0.166 | 0.15168 | 0.183434 | 9.559463 | 8.69505 | 10.603981 |
| 2020 | 1.390973 | 1.12717 | 1.69348 | 0.167865 | 0.152748 | 0.186364 | 9.701837 | 8.786979 | 10.80654 |
| 2021 | 1.363928 | 1.099346 | 1.663296 | 0.16355 | 0.148162 | 0.182594 | 9.468342 | 8.540527 | 10.624259 |
| 2022 | 1.328471 | 1.071513 | 1.617073 | 0.159403 | 0.144209 | 0.180837 | 9.213212 | 8.299906 | 10.379368 |
| 2023 | 1.306703 | 1.050892 | 1.590811 | 0.156226 | 0.14071 | 0.179061 | 9.038333 | 8.108122 | 10.248292 |
| 2024 | 1.284942 | 1.030273 | 1.564559 | 0.153053 | 0.137215 | 0.17729 | 8.863551 | 7.916407 | 10.117358 |
| 2025 | 1.270266 | 1.016383 | 1.547288 | 0.150797 | 0.134844 | 0.175905 | 8.74029 | 7.786785 | 10.019357 |
| 2026 | 1.255576 | 1.002482 | 1.530001 | 0.148537 | 0.132469 | 0.174517 | 8.616836 | 7.656986 | 9.921151 |
| 2027 | 1.24086 | 0.988559 | 1.512683 | 0.146271 | 0.130088 | 0.17312 | 8.493023 | 7.526871 | 9.822541 |
| 2028 | 1.226114 | 0.974613 | 1.495329 | 0.143995 | 0.127699 | 0.171712 | 8.368797 | 7.396398 | 9.72345 |
| 2029 | 1.211347 | 0.96065 | 1.477951 | 0.141713 | 0.125305 | 0.170296 | 8.244269 | 7.265664 | 9.624 |
| 2030 | 1.196564 | 0.946674 | 1.460553 | 0.139427 | 0.122906 | 0.168875 | 8.119521 | 7.134743 | 9.524285 |
| **South Asia** | | | | | | | | | |
| 1992 | 2.255613 | 1.876957 | 2.652743 | 1.264879 | 1.052324 | 1.486175 | 68.229256 | 56.700715 | 80.202776 |
| 1993 | 2.255558 | 1.874095 | 2.638184 | 1.262986 | 1.04894 | 1.480382 | 68.173864 | 56.564619 | 80.015953 |
| 1994 | 2.234127 | 1.881557 | 2.609341 | 1.250997 | 1.057406 | 1.460773 | 67.593517 | 57.112259 | 79.047034 |
| 1995 | 2.247848 | 1.902121 | 2.61568 | 1.262587 | 1.070028 | 1.465043 | 68.154109 | 57.7113 | 79.238221 |
| 1996 | 2.262946 | 1.919326 | 2.647394 | 1.272713 | 1.081606 | 1.486924 | 68.779036 | 58.362889 | 80.44704 |
| 1997 | 2.291817 | 1.959692 | 2.650506 | 1.288224 | 1.10655 | 1.486318 | 69.699978 | 59.742509 | 80.507179 |
| 1998 | 2.33314 | 2.008467 | 2.693278 | 1.308183 | 1.130698 | 1.501797 | 70.842855 | 61.102525 | 81.481909 |
| 1999 | 2.386187 | 2.070526 | 2.733873 | 1.33228 | 1.158759 | 1.522912 | 72.171663 | 62.691083 | 82.594444 |
| 2000 | 2.405632 | 2.085241 | 2.755258 | 1.33533 | 1.161988 | 1.527849 | 72.409226 | 62.892471 | 82.94638 |
| 2001 | 2.420327 | 2.099979 | 2.77043 | 1.334104 | 1.156697 | 1.522013 | 72.361629 | 62.691534 | 82.624946 |
| 2002 | 2.436945 | 2.125796 | 2.786686 | 1.335612 | 1.167084 | 1.521806 | 72.409139 | 63.197434 | 82.646497 |
| 2003 | 2.446631 | 2.127411 | 2.796207 | 1.333978 | 1.165217 | 1.516286 | 72.305448 | 63.107768 | 82.327041 |
| 2004 | 2.447733 | 2.135893 | 2.787264 | 1.326961 | 1.165989 | 1.508609 | 71.974674 | 63.125733 | 82.007467 |
| 2005 | 2.483323 | 2.158014 | 2.826068 | 1.33656 | 1.165609 | 1.519949 | 72.533471 | 63.121011 | 82.608635 |
| 2006 | 2.49711 | 2.18512 | 2.856589 | 1.333737 | 1.171947 | 1.519584 | 72.313227 | 63.46222 | 82.570069 |
| 2007 | 2.52967 | 2.21715 | 2.880787 | 1.338583 | 1.179195 | 1.513698 | 72.531696 | 63.751164 | 82.301319 |
| 2008 | 2.557915 | 2.24352 | 2.916546 | 1.337891 | 1.172654 | 1.520731 | 72.55007 | 63.491906 | 82.700147 |
| 2009 | 2.586501 | 2.239778 | 2.978941 | 1.336111 | 1.156631 | 1.53327 | 72.504065 | 62.710948 | 83.361968 |
| 2010 | 2.614887 | 2.252484 | 3.006682 | 1.333952 | 1.153852 | 1.527361 | 72.398015 | 62.532768 | 83.166373 |
| 2011 | 2.632573 | 2.255359 | 3.056068 | 1.329749 | 1.145672 | 1.538293 | 72.145234 | 61.957654 | 83.625904 |
| 2012 | 2.664243 | 2.268898 | 3.083137 | 1.331046 | 1.135853 | 1.540078 | 72.206113 | 61.447925 | 83.727669 |
| 2013 | 2.730195 | 2.30897 | 3.180896 | 1.347506 | 1.142284 | 1.568751 | 73.111029 | 61.686784 | 85.467088 |
| 2014 | 2.740287 | 2.261149 | 3.223188 | 1.335653 | 1.102771 | 1.567416 | 72.486143 | 59.68458 | 85.330399 |
| 2015 | 2.773425 | 2.223856 | 3.306329 | 1.333195 | 1.069369 | 1.594133 | 72.355212 | 57.883994 | 86.81791 |
| 2016 | 2.851377 | 2.285759 | 3.397201 | 1.352969 | 1.081341 | 1.611866 | 73.394621 | 58.400759 | 87.797732 |
| 2017 | 2.934057 | 2.354166 | 3.4844 | 1.377982 | 1.110528 | 1.638921 | 74.737916 | 59.801359 | 89.27921 |
| 2018 | 2.936786 | 2.309477 | 3.527221 | 1.365472 | 1.08398 | 1.635177 | 74.064329 | 58.353248 | 89.150893 |
| 2019 | 2.953403 | 2.271979 | 3.610178 | 1.359041 | 1.048841 | 1.660568 | 73.734452 | 56.525885 | 90.572698 |
| 2020 | 3.047644 | 2.32156 | 3.769924 | 1.382239 | 1.052976 | 1.706575 | 75.01469 | 56.795792 | 93.103562 |
| 2021 | 3.079166 | 2.335692 | 3.806293 | 1.37679 | 1.05198 | 1.701701 | 74.700083 | 56.684847 | 92.828402 |
| 2022 | 3.091125 | 2.302286 | 3.886162 | 1.376413 | 1.034566 | 1.70354 | 74.866411 | 55.678475 | 93.980232 |
| 2023 | 3.124918 | 2.297399 | 3.968001 | 1.378194 | 1.023388 | 1.715437 | 75.025832 | 55.053878 | 94.989601 |
| 2024 | 3.158802 | 2.292578 | 4.049939 | 1.38006 | 1.012262 | 1.727432 | 75.189226 | 54.431722 | 96.00346 |
| 2025 | 3.186311 | 2.28976 | 4.116884 | 1.383412 | 1.005753 | 1.73876 | 75.392311 | 54.008769 | 96.917647 |
| 2026 | 3.213861 | 2.286976 | 4.183874 | 1.38679 | 0.999255 | 1.750122 | 75.5968 | 53.586585 | 97.833431 |
| 2027 | 3.241434 | 2.284218 | 4.25089 | 1.390173 | 0.992757 | 1.76149 | 75.801646 | 53.164583 | 98.749563 |
| 2028 | 3.268998 | 2.281465 | 4.317889 | 1.393534 | 0.986243 | 1.772833 | 76.005754 | 52.742093 | 99.664691 |
| 2029 | 3.296568 | 2.278728 | 4.384888 | 1.396893 | 0.979725 | 1.784173 | 76.209935 | 52.319601 | 100.579755 |
| 2030 | 3.324209 | 2.27605 | 4.451971 | 1.400294 | 0.973231 | 1.795564 | 76.416108 | 51.898274 | 101.497113 |
| **Southeast Asia** | | | | | | | | | |
| 1992 | 0.809189 | 0.656966 | 0.980311 | 0.356451 | 0.291621 | 0.428062 | 19.406757 | 15.846623 | 23.366629 |
| 1993 | 0.826396 | 0.67203 | 1.004207 | 0.360335 | 0.295729 | 0.43407 | 19.629421 | 16.075802 | 23.693224 |
| 1994 | 0.836552 | 0.680577 | 1.022766 | 0.362492 | 0.298247 | 0.440346 | 19.752005 | 16.219873 | 24.012145 |
| 1995 | 0.851967 | 0.694123 | 1.043914 | 0.367112 | 0.30306 | 0.444823 | 20.006497 | 16.468999 | 24.277554 |
| 1996 | 0.862215 | 0.70181 | 1.056966 | 0.369632 | 0.303629 | 0.447033 | 20.155673 | 16.494294 | 24.4183 |
| 1997 | 0.859272 | 0.702526 | 1.054112 | 0.36761 | 0.302699 | 0.443789 | 20.044272 | 16.473947 | 24.238085 |
| 1998 | 0.869206 | 0.70742 | 1.072313 | 0.369922 | 0.304624 | 0.447093 | 20.160185 | 16.567559 | 24.418848 |
| 1999 | 0.895248 | 0.725966 | 1.103904 | 0.377512 | 0.310574 | 0.454156 | 20.591296 | 16.895512 | 24.836602 |
| 2000 | 0.902981 | 0.727339 | 1.113285 | 0.37806 | 0.31 | 0.458016 | 20.630832 | 16.868384 | 25.060084 |
| 2001 | 0.897162 | 0.723162 | 1.105477 | 0.373728 | 0.307564 | 0.454385 | 20.376271 | 16.72819 | 24.801024 |
| 2002 | 0.89371 | 0.719424 | 1.108997 | 0.36973 | 0.303939 | 0.448102 | 20.143485 | 16.503023 | 24.476327 |
| 2003 | 0.887685 | 0.720323 | 1.092168 | 0.365523 | 0.302376 | 0.439431 | 19.905721 | 16.419212 | 23.995333 |
| 2004 | 0.875963 | 0.71577 | 1.065781 | 0.359169 | 0.299459 | 0.429215 | 19.548893 | 16.243027 | 23.445555 |
| 2005 | 0.858461 | 0.708416 | 1.036276 | 0.350919 | 0.294809 | 0.417506 | 19.08876 | 15.986909 | 22.747975 |
| 2006 | 0.850775 | 0.703145 | 1.024023 | 0.344147 | 0.291316 | 0.408951 | 18.724818 | 15.825179 | 22.231407 |
| 2007 | 0.852792 | 0.711242 | 1.02496 | 0.340666 | 0.287889 | 0.40437 | 18.535094 | 15.615556 | 21.996026 |
| 2008 | 0.853283 | 0.711451 | 1.028325 | 0.336918 | 0.28598 | 0.398916 | 18.33096 | 15.536548 | 21.744777 |
| 2009 | 0.850764 | 0.707456 | 1.018669 | 0.332338 | 0.279092 | 0.394673 | 18.080549 | 15.170921 | 21.509199 |
| 2010 | 0.851228 | 0.71219 | 1.01579 | 0.328297 | 0.27761 | 0.387233 | 17.86252 | 15.093717 | 21.075702 |
| 2011 | 0.858918 | 0.716703 | 1.027449 | 0.328055 | 0.276747 | 0.386203 | 17.851152 | 15.034579 | 21.067628 |
| 2012 | 0.86068 | 0.717847 | 1.027661 | 0.32573 | 0.274066 | 0.386363 | 17.718951 | 14.884316 | 21.038602 |
| 2013 | 0.865369 | 0.713318 | 1.039359 | 0.324891 | 0.271487 | 0.384454 | 17.66764 | 14.768262 | 20.902988 |
| 2014 | 0.875114 | 0.724033 | 1.051533 | 0.325356 | 0.272021 | 0.387905 | 17.690915 | 14.77198 | 21.139594 |
| 2015 | 0.890403 | 0.737271 | 1.075161 | 0.326995 | 0.273143 | 0.389142 | 17.778327 | 14.822328 | 21.199256 |
| 2016 | 0.904055 | 0.743392 | 1.089205 | 0.327984 | 0.270269 | 0.392547 | 17.832905 | 14.694763 | 21.38876 |
| 2017 | 0.914163 | 0.755645 | 1.102894 | 0.327885 | 0.272872 | 0.391082 | 17.828415 | 14.81525 | 21.274711 |
| 2018 | 0.926202 | 0.756665 | 1.126817 | 0.3281 | 0.270407 | 0.392966 | 17.848416 | 14.700492 | 21.375376 |
| 2019 | 0.942498 | 0.763311 | 1.160309 | 0.32951 | 0.269099 | 0.400759 | 17.937063 | 14.684806 | 21.812559 |
| 2020 | 0.93544 | 0.744702 | 1.157362 | 0.323893 | 0.262894 | 0.397386 | 17.625265 | 14.297896 | 21.619079 |
| 2021 | 0.947772 | 0.748862 | 1.180886 | 0.325083 | 0.260811 | 0.398789 | 17.723221 | 14.21163 | 21.714418 |
| 2022 | 0.964292 | 0.757039 | 1.19771 | 0.321915 | 0.264014 | 0.397271 | 17.688259 | 14.402751 | 21.780653 |
| 2023 | 0.974467 | 0.758034 | 1.214758 | 0.320216 | 0.262936 | 0.397554 | 17.651914 | 14.356087 | 21.851111 |
| 2024 | 0.984619 | 0.759014 | 1.231774 | 0.318508 | 0.26185 | 0.397823 | 17.615069 | 14.309084 | 21.920916 |
| 2025 | 0.992291 | 0.759659 | 1.244968 | 0.317403 | 0.261204 | 0.398192 | 17.587971 | 14.275412 | 21.964938 |
| 2026 | 0.999954 | 0.760299 | 1.25815 | 0.316295 | 0.260556 | 0.398559 | 17.560826 | 14.2417 | 22.008906 |
| 2027 | 1.007617 | 0.760937 | 1.271333 | 0.315191 | 0.25991 | 0.39893 | 17.533863 | 14.208106 | 22.053115 |
| 2028 | 1.015261 | 0.761561 | 1.284491 | 0.31408 | 0.25926 | 0.399294 | 17.506658 | 14.174317 | 22.09703 |
| 2029 | 1.022879 | 0.762168 | 1.297614 | 0.312961 | 0.258602 | 0.399645 | 17.479028 | 14.140216 | 22.140399 |
| 2030 | 1.0305 | 0.762777 | 1.310737 | 0.311844 | 0.257948 | 0.4 | 17.451619 | 14.106277 | 22.184026 |

ASR: age-standardized rate; ASIR: age-standardized incidence rate, ASMR: age-standardized mortality rate, DALYs: disability-adjusted life-years.

Supplementary Table 12**.** Residual analysis of the Nordpred APC model for case number and ASR of lip and oral cavity cancer incidence, deaths, and DALYs.

| **Location** | **Average relative error (%)** | | | | | |
| --- | --- | --- | --- | --- | --- | --- |
|  | Incidence | | Deaths | | DALYs | |
|  | Number | ASR | Number | ASR | Number | ASR |
| East Asia | 0.00520 | 0.0000376 | 0.01716 | 0.0001376 | 0.00036 | 0.0000024 |
| High-income Asia Pacific | 0.02130 | 0.0000204 | 0.16739 | 0.0001385 | 0.00233 | 0.0000027 |
| South Asia | 0.00141 | 0.0000081 | 0.00322 | 0.0000151 | 0.00004 | 0.0000003 |
| Southeast Asia | 0.01053 | 0.0000260 | 0.02822 | 0.0000730 | 0.00053 | 0.0000014 |

APC: age-period-cohort; ASR: age-standardized rate; DALYs: disability-adjusted life-years.
